# Unlocking the Sublime: A Review of Native Australian *Citrus* Species

**DOI:** 10.3390/foods14142425

**Published:** 2025-07-09

**Authors:** Joel B. Johnson, Natasha L. Hungerford, Yasmina Sultanbawa, Michael E. Netzel

**Affiliations:** 1 Centre for Nutrition and Food Sciences, Queensland Alliance for Agriculture and Food Innovation (QAAFI), The University of Queensland, Health and Food Sciences Precinct, Coopers Plains, Brisbane, QLD 4108, Australia; y.sultanbawa@uq.edu.au; 2 Centre for Animal Science, Queensland Alliance for Agriculture and Food Innovation (QAAFI), The University of Queensland, Health and Food Sciences Precinct, Coopers Plains, Brisbane, QLD 4108, Australia; n.hungerford@uq.edu.au

**Keywords:** citrus, horticulture, indigenous species, composition, bioactive properties, traditional use

## Abstract

Citrus fruit are well-known for their characteristic flavour and nutritional value. Global citrus production has increased by 528% between 1961 and 2021, and in Australia, citrus is the most exported fresh fruit product by volume. There are six described *Citrus* species endemic to Australia: *C. australasica* (Australian finger lime), *C. australis* (round lime), *C. garrawayi* (Mount White lime), *C. glauca* (desert lime), *C. gracilis* (Humpty Doo lime), and *C. inodora* (Russell River lime). Australian *Citrus* possess unique flavours, aromas, and phytochemical profiles, suggesting a potential use as novelty crops and/or ‘functional foods’. Furthermore, the native Australian *Citrus* germplasm is a valuable source of desirable traits in citrus breeding, including drought, cold, heat, salinity, and disease resistance. These may help solve some challenges facing citrus growers globally, including disease, a declining soil quality, changing climates, and narrowing profit margins. However, many Australian citrus species’ nutritional value, chemical composition, and bioactive properties remain unknown. This review focuses on these under-investigated native *Citrus* species, their distribution, production, physiology, disease tolerance, traditional use, taxonomy, flavour, nutritional composition, bioactivity, and commercial production. It concludes with a perspective on the future of these native species in the Australian and global citrus context.

## 1. Introduction

### 1.1. Native Australian Flora and Foodplants

Approximately 18,700 species, or 93% of all Australian flowering plants, are believed to be endemic to Australia [1] and hence do not naturally occur in any other region of the world. Despite this wealth of biodiversity, many native Australian species are under-studied or have never been studied from a scientific perspective. These include many edible plants which have traditionally been utilised by Indigenous Australians.

However, there are numerous beneficial traits found in indigenous Australian food plants. Firstly, they are naturally adapted to Australian climatic conditions, which should reduce fertiliser and irrigation requirements. Some bushfoods contain higher levels of health-beneficial compounds, allowing them to be classified as ‘functional foods’—foods which provide health benefits in addition to their basic nutritional value and thus attract significant price premiums [2]. As an example, the Kakadu plum (*Terminalia ferdinandiana*) contains 20–100 times more vitamin C than oranges [3,4]. Furthermore, many species provide unique organoleptic properties and flavours not found in other foodplants [5], which can attract price premiums and support the uptake in boutique restaurants.

This review focuses on one specific group of under-investigated native Australian foodplants—the *Citrus* genus. It begins with an overview of the genus and the nutritional value of citrus fruit before summarising global and Australian citrus production. The main part of this review details the current state of knowledge for all six native Australian *Citrus* species. It concludes with a perspective on the future of these native species in the Australian and global citrus context.

### 1.2. Citrus: Classification

The Rutaceae family contains over 2000 species, many of which are trees or shrubs with aromatic flowers and leaves [6]. *Citrus* is the best-known and most commercially significant genus, being classified in the Aurantioideae subfamily, Citreae tribe, and Citrinae subtribe (see Table 1). The Citrinae subtribe (‘true citrus’ species) is characterised by the pulp vesicles found in the fruit [7]. These vesicles grow from the dorsal wall of the locule into the locular cavity, eventually forming sac structures comprising large, thin-walled cells which hold the juice.

**Figure 1:**
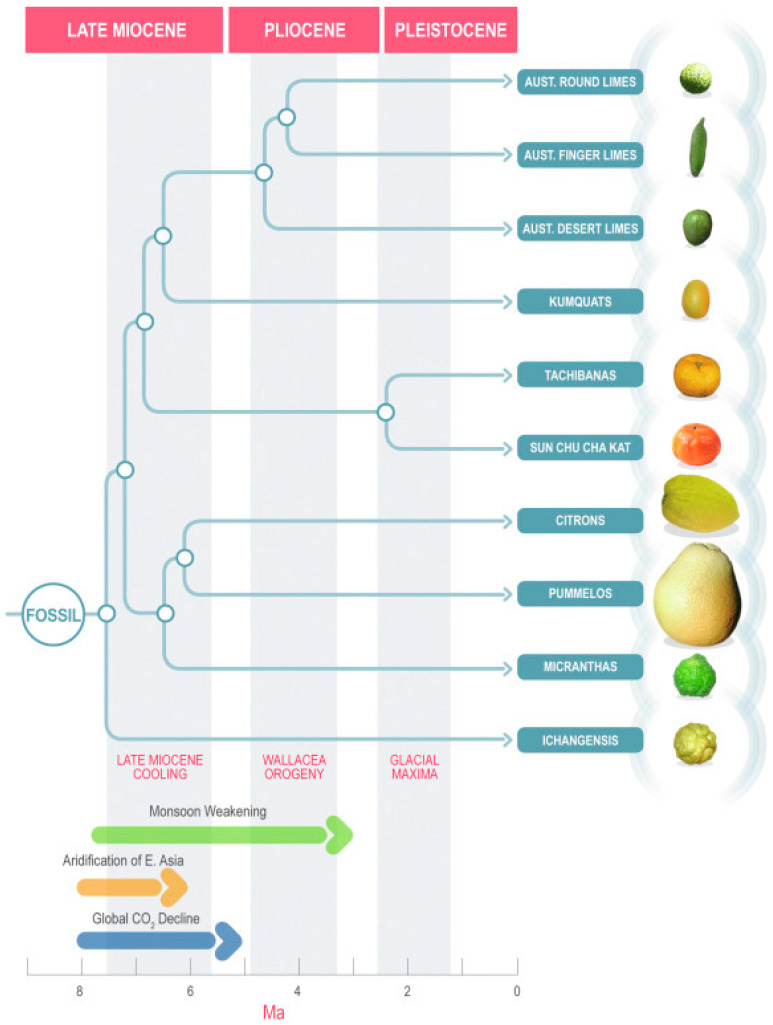
A potential chronogram of citrus speciation, as proposed by Wu et al. [11]. Reproduced from Talon et al. [20] with permission from the publishers. Ma stands for mega annum.

There is no clear consensus on the exact number of *Citrus* species due to the hundreds of hybrids found in this genus. One commonly used classification system proposes 16 species [8], while Mabberley [9] proposed only 3 *Citrus* species (*C. medica*, *C. maxima,* and *C. reticulata*), alongside 4 hybrid groups. At the other extreme, Tanaka [10] included 156 *Citrus* species in his classification system. More recently, Wu et al. [11] used whole-genome sequencing to propose 10 ancestral (‘true’) *Citrus* species (Figure 1); three of which were from Australia (*C. australis*, *C. australasica,* and *C. glauca*). While numerous phylogenetic studies have focused on the *Citrus* taxonomy in recent years [11,12,13], the results are somewhat contrasting, leaving the exact classifications still in flux. For nomenclature, this review uses the taxonomic system of Zhang and Mabberley [14], which retains binomial names for pure ancestral species.

The *Citrus* genus encompasses all well-known commercial citrus varieties such as orange (*Citrus × aurantium* var. *sinensis*), mandarin (*C. reticulata*), lemon (*C. × limon*), and grapefruit (*C. × paradisi*)—as well as six *Citrus* species which are native to Australia. The Australian species were traditionally separated into a ‘*Microcitrus*’ genus (*C. australasica*, *C. australis*, *C. garrawayi,* and *C. inodora*) and ‘*Eremocitrus*’ genera (*C. glauca*) by Swingle [15] but were reunited in the *Citrus* genus by Mabberley [16]. The heterozygosity levels of all of the Australian species are less than 1% [17], confirming that they can be considered ‘pure’ or ‘true’ citrus species [11].

**Table 1:** The traditional classification of the Aurantioideae subfamily following Swingle, adapted from Luro et al. [18] and Bayer et al. [19].

Subfamily	Tribe	Subtribe	Group	Genus
Aurantioideae	Clauseneae	Micromelinae	-	*Micromelum*
		Clauseninae	-	*Clausena* *Glycosmis* *Murraya*
		Merrilliinae	-	*Merrillia*
	Citreae	Triphasiinae	-	*Luvunga* *Merope* *Monanthocitrus* *Oxanthera Pamburus* *Paramignya* *Triphasia* *Wenzelia*
		Balsamocitrinae	-	*Aegle* *Aeglopsis* *Afraegle* *Balsamocitrus* *Feronia* *Feroniella* *Swinglea*
		Citrinae	Group A (‘Primitive Citrus)	*Burkillanthus* *Hesperethusa Limnocitrus* *Pleiospermium* *Severinia*
			Group B (‘Near Citrus’)	*Atalantia Citropsis*
			Group C (‘True Citrus’)	*Citrus* *Clymenia* *Eremocitrus* *Fortunella* *Microcitrus* *Poncirus*

### 1.3. Commercial Citrus Production: A Global Overview

Citrus is the most widely grown tree fruit crop used for direct consumption [21,22], making its global significance indisputable. Furthermore, citrus was identified by the International Treaty on Plant Genetic Resources for Food and Agriculture as one of thirty-five food crops essential to the conservation and development of crop diversity [23]. From a nutritional aspect, citrus fruit contains an abundance of nutrients and potentially health-beneficial constituents [24]. Highlighting this, orange juice has the highest nutrient density (quantity of nutrients per calorie) of any fruit juice [25].

**Figure 2:**
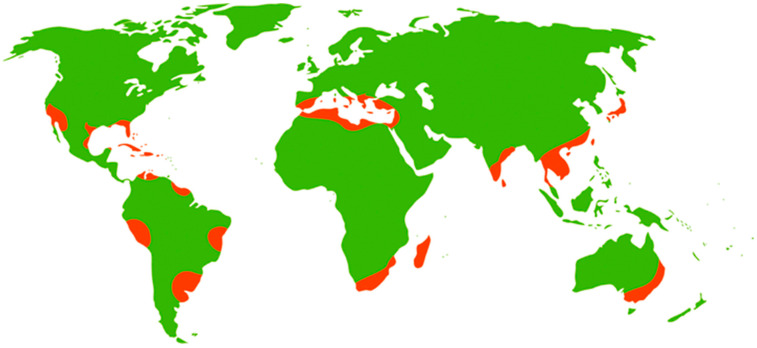
Major citrus-producing regions across the world (shown in red). Reproduced from Liu et al. [26] with permission from the publisher.

The majority of citrus production occurs in subtropical regions (Figure 2), between approximately 35° N and 35° S [26]. As of 2021, the largest global producers of citrus include China (approximately 43.6 million tonnes per annum), Brazil (17.4 MT p.a.), India (10.8 MT p.a.), Mexico (5.8 MT p.a.), Spain (5.7 MT p.a.), and the United States (5.5 MT p.a.) [27].

The global citrus production has increased significantly over the last six decades, with a 528% increase in the total production between 1961 and 2021 (Table 2). The largest increase (1380%) was seen for tangerine, mandarin, and clementine production, followed by a marked increase (845%) in ‘other citrus’ crops. On the other hand, orange and pomelo/grapefruit production has grown more moderately (342–373%) over these 60 years (Table 2).

Around two-thirds of all citrus fruit is eaten fresh, with the remainder processed before consumption—orange juice being the main product [26]. Other processed products include preserves, marmalades, and jellies. Citric acid, pectin, and essential oils can be extracted from citrus by-products (peel and low-grade fruit) [28].

### 1.4. Commercial Citrus Production in Australia

Australia is a small citrus producer on the global stage, harvesting 760,000 tonnes in 2022, with a farmgate value of AUD 910 million [29]. Citrus accounts for 1.5% of the gross value of Australian farm production but 8% of the gross value of Australian horticultural production [30].

**Figure 3:**
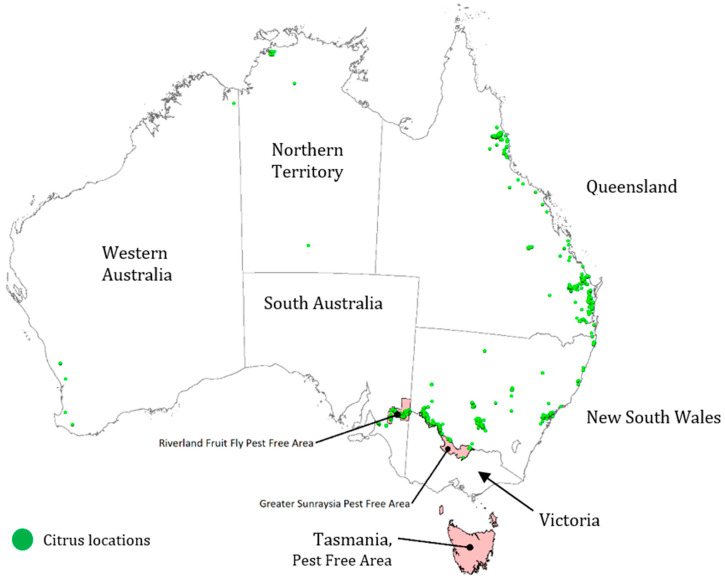
Citrus-producing regions in Australia (shown in green). The pink areas are designated pest-free (fruit fly exclusion) zones, where significant portions of citrus production take place. Reproduced from Hogan et al. [30] under a Creative Commons 4.0 license.

Furthermore, the citrus industry is the largest exporter of fresh fruit in Australia, exporting around AUD 520 million worth of produce in 2019–2020 [30]. Exports have more than doubled between 2009–2010 and 2019–2020, with a particularly sharp rise after 2015 [30]. The major export markets are in Asia, including China, Japan, Hong Kong, and Malaysia. Around 71% of fruit is sold fresh (internationally or domestically), with the remainder destined for processing [29]. Citrus production is concentrated in four major regions (Figure 3): the Riverina (southwest NSW), Riverland (eastern South Australia), Sunraysia (northwest Victoria), and the eastern Queensland coast. Western Australia and the Northern Territory are minor producers.

The most common citrus varieties are oranges (501,072 tonnes p.a.), followed by mandarins (181,893 t. p.a.), lemons/limes (65,920 t. p.a.), and grapefruit (11,190 t. p.a.) [29]. Minor or niche citrus varieties include tangelos, tangerines (usually counted under mandarins in statistical data), pomelos (usually counted under grapefruit), and Australian finger limes (*C. australasica*). *C. australasica* are the only native Australian *Citrus* species currently grown at any commercial scale (10–20 t. p.a.), although they are primarily restricted to boutique applications.

### 1.5. Challenges to the Australian Citrus Industry: The Role of Native Citrus

Despite its recent rapid growth, the Australian citrus industry faces numerous challenges. Disease is one of the major challenges in the immediate term, including the fungal disease anthracnose (*Colletotrichum* spp.) [31], aphids, leafminers, and gall wasps. The most serious disease is huanglongbing, caused by the bacteria *Candidatus liberibacter asiaticus*, which produces uneven leaf mottling and eventually kills the tree [32]. Although not yet found in Australia, huanglongbing has devastated citrus industries worldwide [33]. However, native Australian *Citrus* species show a resistance or tolerance against huanglongbing [34] and thus could be safely grown (or used as rootstocks).

**Figure 4:**
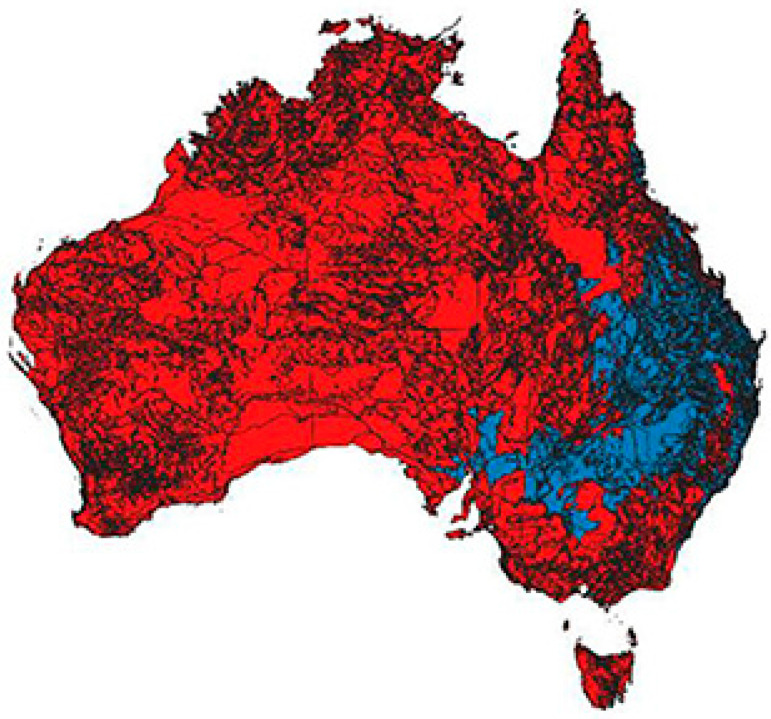
The modelled environmental range (in blue) which may be suitable for growing native *Citrus* species, under current climatic conditions. Adapted from Canning [36] under the Creative Commons 4.0 license.

A longer-term challenge is climate change. Citrus are highly sensitive to environmental factors such as humidity and temperature [35]; hence, it is crucial to grow citrus varieties/species which are more tolerant of water stress, salinity, flooding, and high/low temperatures. Warmer temperatures may also increase the spread of existing pests and diseases. Being already adapted to Australian environmental conditions, some native *Citrus* are more water-efficient and tolerant of temperature extremes. Furthermore, modelling by Canning [36] suggests native *Citrus* (particularly *C. glauca*) could be cultivated across drier and/or warmer portions of eastern Australia where citrus is not currently grown (particularly central and western Queensland, see Figure 4).

Finally, Australian citrus growers face an increasing economic challenge which threatens their long-term sustainability. Citrus production is a low-margin crop, and there is significant competition from cheaper international produce. Australian growers must differentiate their produce based on its higher quality, better nutritional value, or other desirable features. Growing novel citrus varieties (such as native Australian *Citrus*) would be one way to ensure a unique product and target high-value markets.

As outlined above, native *Citrus* species may hold the key to solving several of the primary challenges confronting the Australian citrus sector. Furthermore, many of these species show other unique characteristics (e.g., flavour and chemical composition), making them worthy of further in-depth investigation. The remainder of this review focuses on native Australian *Citrus* species, firstly covering their distribution, morphology, physiology, and disease tolerance, before moving on to their traditional use, taxonomy, flavour, and aroma, and the chemical composition of the pulp, peel, leaves, and seeds. It finishes with a perspective on the current status and prospects for commercial production, along with the anticipated future research directions for these species.

## 2. Introduction to Native Australian Citrus Species

There are six described species of *Citrus* which are native to Australia: *Citrus australasica* F.Muell., *C. australis* (A.Cunn. ex Mudie) Planch., *C. garrawayi* F.M.Bailey, *C. glauca* (Lindl.) Burkill, *C. gracilis* Mabb., and *C. inodora* F.M.Bailey. This is the largest number of citrus species which are indigenous to a single country [37]. Each of these are covered in detail in the following sections. Characteristic features of most native Australian citrus include their exceptionally short juvenile stages, rapid fruit maturation, resistance to nematodes and many diseases, and zygotic embryony (in contrast to the asexual nucellar embryony seen in most other *Citrus* species) [38,39].

*C. australis* was the first to be collected by European explorers—by the botanist Allan Cunningham from the Brisbane River in 1827. Its common names include the Gympie lime, Australian lime, Australian round lime, native lime, native orange, and Dooja (an Aboriginal name). Although live plants were sent to the Kew Botanical Gardens as early as 1829 [40], it was not widely propagated.

The second species to be described was *Citrus australasica* in 1858 [41]. It is undisputably the best-known and most researched of all native Australian *Citrus* species.

*Citrus glauca* is the only known xerophyte in the *Citrus* genus [42], commonly known as the Australian desert lime, lime bush, bush lime, wild lime, native lime, wild kumquat (cumquat), or desert kumquat [16]. First collected by Robert Brown on Flinders’ voyage (1801–1803) [43], it was subsequently recorded during Ludwig Leichhardt’s overland expedition [44], before the type specimen was collected by the surveyor Sir Thomas Mitchell in 1846 [16].

*Citrus inodora* is commonly called the Russell River lime, North Queensland lime, large-leaf Australian wild lime, or Maiden’s Australian wild lime (for *C. inodora* var. *maideniana*). Despite its remote distribution, it has been propagated widely since its description in 1889.

*Citrus garrawayi*, described in 1904, is known as the Mount White lime, Garraway’s lime, Garraway’s Australian wild lime, or thick-skinned finger lime. Although now considered a ‘least concern’ (2024), it was previously listed as ‘rare’ under the Queensland Nature Conservation Act 1992 [45].

*Citrus gracilis*, also called the Humpty Doo lime or Kakadu lime, was first collected in 1971 and described in 1998. The scientific literature on this species is very limited.

## 3. Distribution

**Figure 5:**
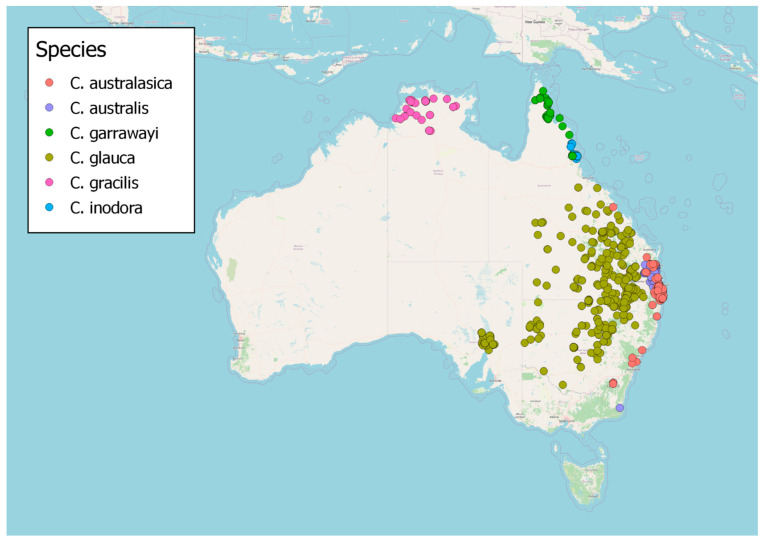
The distribution of the six native Australian *Citrus* species, plotted using record data sourced from the Atlas of Living Australia (https://ala.org.au; accessed 17 June 2024). Figure by Joel Johnson, reproduced under the Creative Commons 4.0 licence (https://doi.org/10.6084/m9.figshare.27001948).

As seen in Figure 5, most Australian citrus are endemic to the eastern coast. *C. glauca* is the only species extending inland, predominantly found in heavy clay brigalow forests of inland Queensland and New South Wales (receiving 580–860 mm of annual rainfall) [46]. Although once prevalent [47], agricultural clearing has led to it becoming threatened in some regions [48]. There is also a disjunct population near Port Augusta in South Australia [42], which is currently classified as vulnerable.

Two species—*C. australasica* and *C. australis*—are found in subtropical rainforests of the Queensland/New South Wales border, particularly on red volcanic soils. *C. australis* has a more restricted range and occurs in lighter, drier rainforests [49], typically araucarian microphyll vine forests, while *C. australasica* tends to occur in complex notophyll vine forests [50]. Both *C. australasica* and *C. australis* are adapted to low soil fertility and heavy rainfall [51].

A further two *Citrus* species are found on the Cape York Peninsula. *C. inodora* is restricted to Mount Bellenden Ker and Mossman, principally occurring in lowland complex mesophyll vine forests on metamorphic soils [50]. Most lowland populations of *C. indora* along the Russell River were lost due to land clearing in the 20th century [52]; the species is currently listed as vulnerable. *C. inodora* is endemic to one of the wettest regions in Australia, receiving up to 8000 mm in annual rainfall. *C. garrawayi* is found further north on Cape York, on complex microphyll and notophyll vine forests and vine thickets [50]. Its type location is Mount White, near Coen.

Finally, *Citrus gracilis* is found from Darwin through to Mataranka in the Northern Territory. *C. gracilis* is widespread across this region but never locally common [53]. It occurs on sandy or gravelly soil [16], in association with *Eucalyptus tetrodonta*, *E. miniata*, *Vitex glabrata,* and/or *Canarium australianum* [53].

## 4. Description

**Figure 6:**
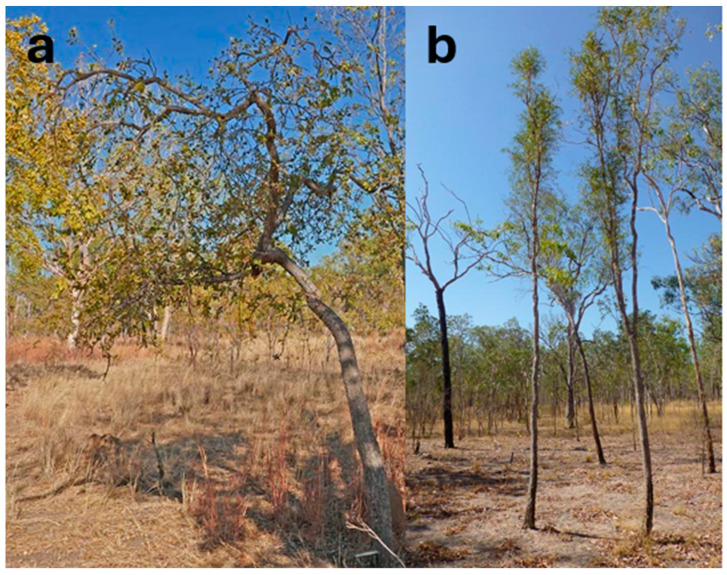
*Citrus gracilis* trees, showing the straggling form (**a**) and the straight form (**b**). Photographs by Michael Saalfeld, used with permission.

Most of the native Australian *Citrus* species are shrubs to small trees. *C. australasica*, *C. glauca*, and *C. inodora* tend to be more straggling and shrub-like, typically reaching a maximum height of 3–5 m [52]. However, *C. glauca* reaches 4–15 m in the wild [42]. Other species (*C. australis* and *C. garrawayi*) are more tree-like, reaching a height of up to 10 m or more [15,54]. *C. gracilis* appears to have two morphological forms (Figure 6): one of a short, straggling tree (4 m), with a rough, irregularly cracked, red–grey bark [16], and the other comprising straighter, taller (10–12 m) trees with a smooth bark and different fruit shapes/locule numbers [55].

All species bear spines along their stems, likely as a defence against herbivores. These typically reach 10–15 mm in length, although they may be longer in some species. Most bear solitary spines; however, *C. inodora* is unique in bearing a pair of spines at its petiole base, unlike any other *Citrus* species [56]. In *C. glauca*, the spines are largest in young saplings; as the tree matures they shrink and often disappear in the higher branches but not in the low-hanging branches [42].

Most Australian *Citrus* have small leaves, with the elliptical leaves of *C. australasica* being the smallest (2.2–2.5 × 1.4–1.5 cm) [57]. Those of *C. australis* and *C. glauca* are also longer (4–5 cm) [49] but are still relatively small. *C. glauca* has unique coriaceous (leathery) foliage, which is dotted with oil glands [49]. The juvenile foliage of *C. garrawayi* is dimorphic and much smaller than mature leaves [58], which reach 2.5–4.5 × 1–2.5 cm and have prominent, oblique veins [59]. Similarly, the juvenile leaves of *C. gracilis* are narrower but grow wider to become lanceolate or oblanceolate (3–6 × 1.1–2 cm) during maturation [16]. *C. inodora* has quite large leaves (8–20 × 4–10 cm) with numerous lateral veins [16]; they are glossy dark green on the upper surface but lighter green on the underside [60]. Very young leaves have serrated margins.

Flowers are generally white (e.g., *C. australis* [61] and *C. garrawayi*) but can range from pink–white (*C. australasica* [61]) to pink/purple–white (*C. gracilis* [16]) as well as from green–white (*C. glauca* [46]) to pale yellow (*C. inodora* [60]). Most are small (up to around 10 mm in diameter).

The ‘*Microcitrus*’ group (sensu Swingle) typically has three locules, with *C. australasica* being 3- or 4-merous [61], *C. australis* being 4- or 5-merous [61], and C. glauca being 3- to 5-merous [62]. *C. inodora* has 6–8 ovary locules [52,60,63], while *C. gracilis* has 8 or 9 locules [16]. Flowers of the ‘*Microcitrus*’ group (sensu Swingle) are mainly produced singly, although sometimes occurring in groups of three in *C. gracilis* [16]. Those of *C. glauca* are sweet-scented [64], while *C. inodora* flowers have very little scent (hence the specific epithet *inodora*). Both male and hermaphroditic (perfect) flowers have been reported from *C. inodora* [60], while further fieldwork is needed to determine if *C. gracilis* is dioecious or if this species produces hermaphrodite and functionally male flowers [16].

Flowering times vary widely, from late winter/early spring (July–September) for *C. glauca* [46], August to September for *C. inodora* [65], spring for *C. australis* [66], and to May and November–December for *C. garrawayi*. The fruit set between April and November for *C. garrawayi* [65], between November and December for *C. glauca* [42,67], across a 5–8 week period between February and September for *C. australasica* [68,69,70], and in December or between March and September for *C. inodora* [65]. The flowering and fruiting of *C. gracilis* appears to be triggered by the monsoonal rainfall in the Northern Territory (usually late spring/early summer). Fruit are reported in September [55], March, and May [53]. The *C. glauca* fruit ripen 10–12 weeks after flowering, which is the shortest fruiting period of any known citrus species [71]. In contrast, *C. australasica* has a more typical fruiting period length of 5 months.

**Figure 7:**
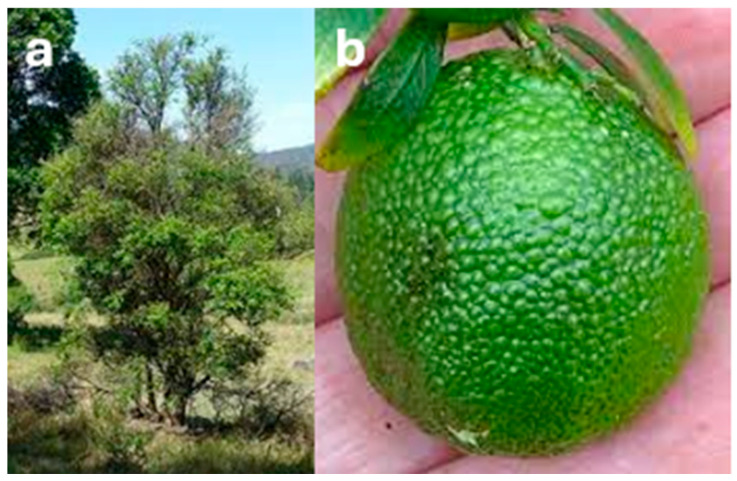
A *Citrus australis* tree (**a**) and fruit (**b**). Photograph by Michael Saalfeld, used with permission.

**Figure 8:**
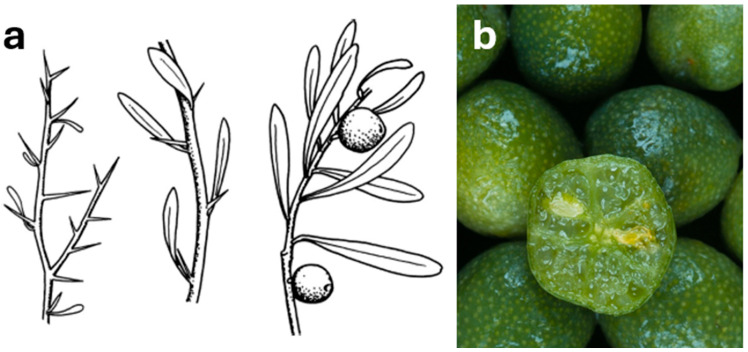
(**a**) Different forms of *Citrus glauca* vegetation, from immature (**left**) to mature (**right**). Note the absence of thorns in the mature vegetation. Illustration by David Mackay, used with permission. © Royal Botanic Gardens and Domain Trust. (**b**) *Citrus glauca* fruit. Photograph reproduced from Wikipedia (CSIRO), under Creative Commons 3.0 license.

Fruit from *C. australis* has the most similar appearance to conventional citrus species (Figure 7), with the dark green, round fruit reaching 5 cm in diameter [49] and typically comprising six segments [72]. *C. glauca* also has round fruit—but they are the size of a grape (1–1.5 cm diameter; Figure 8b), making them the smallest of any citrus species—with the exception of the kumquat (‘*Fortunella*’ sensu Swingle) [73]. The fruit have three locules [16] and are typically seedless [42] and thin-skinned, turning from green to light yellow during ripening [67]. Non-fruiting forms of *C. glauca* also exist [16]. Both *C. glauca* [74] and *C. gracilis* [16] are believed to primarily spread through suckering.

**Figure 9:**
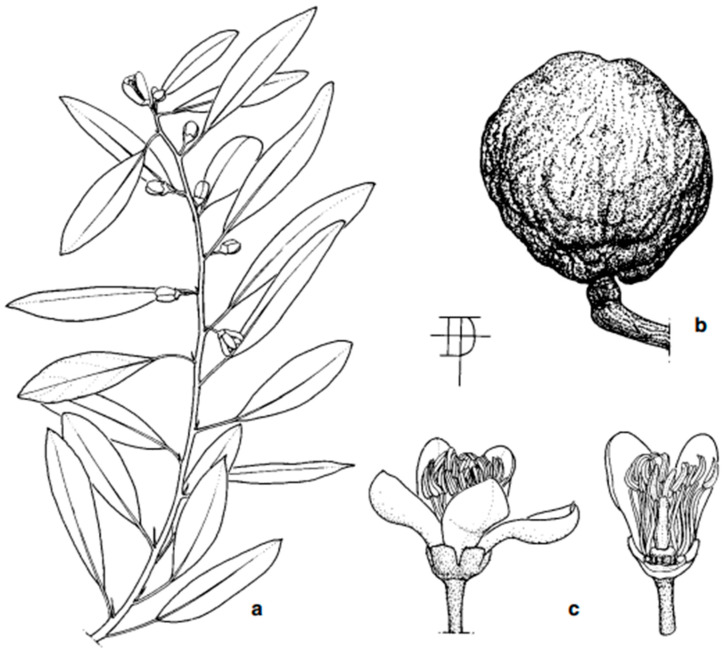
Diagrams of *Citrus gracilis*, reproduced from Mabberley [16] with permission from the illustrator (Donald Fortescue). (**a**) A flowering twig. (**b**) Fruit. (**c**) Functionally male flowers.

**Figure 10:**
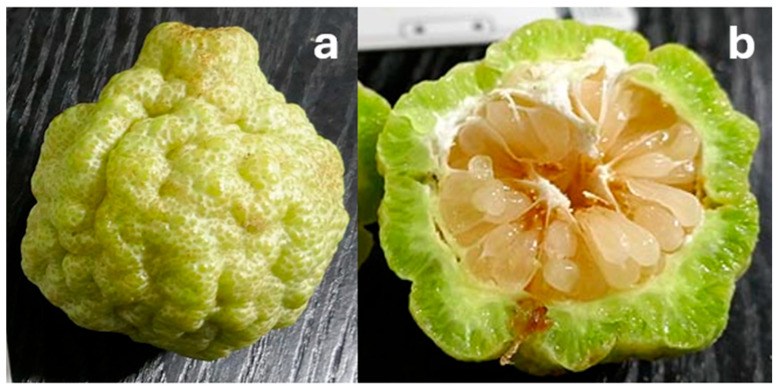
Photographs of mature *Citrus gracilis* fruit, showing the exterior (**a**) and a latitudinal cross-section (**b**). Photographs by Michael Saalfeld, used with permission.

*C. gracilis* has never been successfully propagated from seeds or cuttings; only small (1 mm diameter), nonviable seeds have been found. Its fruit are green and globose to pyriform in shape (Figure 9b and Figure 10) and are the largest of any native Australian *Citrus* (8–10 cm diameter). The vesicles are also much larger.

**Figure 11:**
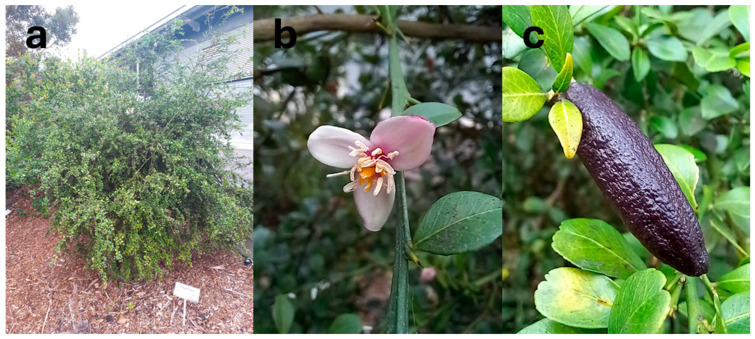
A young *Citrus australasica* plant (‘Ricks Red’ finger lime), showing the (**a**) shrub, (**b**) flower, and (**c**) fruit. Photographs by Joel Johnson, reproduced under the Creative Commons 4.0 licence (https://doi.org/10.6084/m9.figshare.27002065).

**Figure 12:**
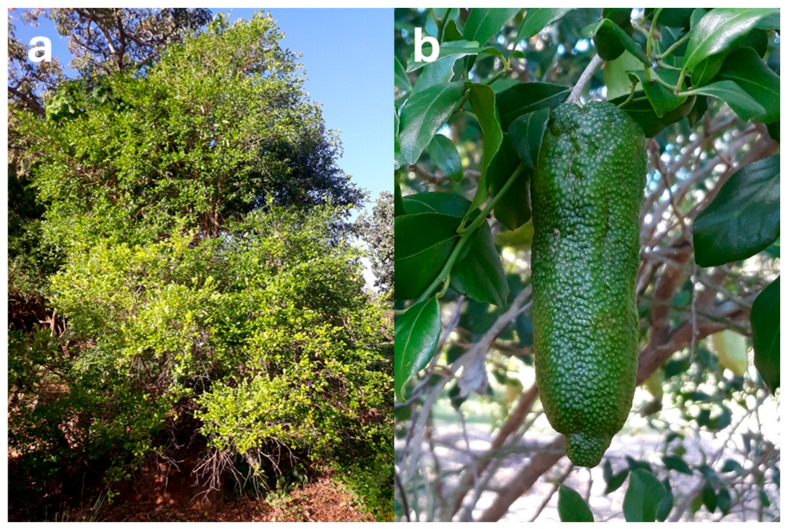
A *Citrus garrawayi* tree (**a**) and fruit (**b**). Figure by Joel Johnson, reproduced under the Creative Commons 4.0 licence (https://doi.org/10.6084/m9.figshare.27002119).

**Figure 13:**
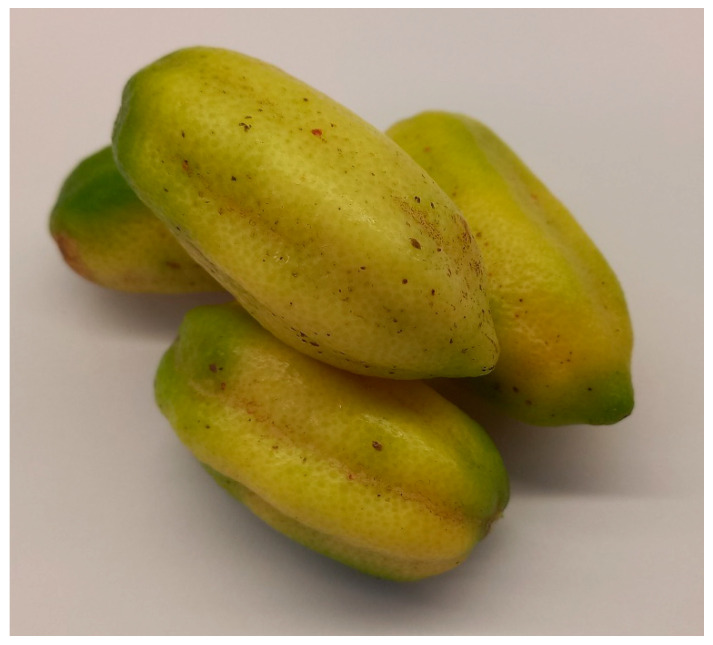
*Citrus inodora* fruit. Photograph by Joel Johnson, Flickr (https://www.flickr.com/photos/195631385@N06/53988291097 (accessed on 1 July 2025)). Used with permission.

The remaining native Australian *Citrus* species have elongated, cylindrical fruit, reaching 7 cm (*C. australasica* and *C. inodora*) to 10 cm in length (*C. garrawayi*). The peel colour of *C. australasica* varies widely from green, red, and yellow to purple–black (Figure 11) [49]. The pulp is contained in small spherical vesicles which are cream/green to deep red in colour [75], giving rise to the common name ‘caviar lime’ [49]. The *C. garrawayi* fruit contains similar, cream to pink subglobose juice vesicles [58], with rounded/triangular seeds reaching 5–7 mm length [76]. The peel is dark green and has a tuberculose appearance (Figure 12b) due to prominent oil glands [59]. *C. inodora* fruit (6–7 × 3 cm) also contain spherical juice vesicles, but the peel is lemon yellow when ripe [49,52] and possesses unique lengthwise ribbing [15] (Figure 13).

## 5. Physiology

### 5.1. Frost and Cold Tolerance

Most *Citrus* species have a limited cold tolerance, growing best between 16 and 32 °C and suffering significant damage from temperatures below −5 °C [77,78]. *C. australasica* and *C. australis* are more frost tolerant than most commercial *Citrus* species, with dieback rates of 51% and 74% reported after −4 °C minimums, respectively [79]. This tolerance is attributed to the proline and proline betaine which accumulate in the leaves [80]. In contrast, *C. inodora* showed a 93% dieback under the same conditions. The seeds of both *C. garrawayi* and *C. inodora* fail to germinate at 15 °C or colder, with *C. garrawayi* showing the poorest cold tolerance of the three Australian citrus species tested [81].

*C. glauca* is one of the most cold-tolerant species known from the *Citrus* genus, again attributed to the accumulation of the high levels of proline, proline betaine, and hydroxyproline betaine in its leaves [80]. The figure of −24 °C widely reported in the literature [66,82,83,84] is likely due to a misinterpretation of the original statement of ‘… *ten or more degrees below freezing Fahrenheit*…’ made by Swingle and Reece [62]. Primary observations have reported *C. glauca* suffering no serious effects at temperatures of −2 to −4 °C [85], −4 °C [67], −8 °C [42], −8.3 °C [73], −8.9 °C [83], below −10 °C [86], and −14 °C [77]. Its cold tolerance is transmitted both as a rootstock [77] and through breeding [83], with *C. glauca* hybrids surviving at −7.2 °C [84] and −10.0 °C [77]. Additionally, it can flower at 0 °C [73]. Based on this, its true cold tolerance likely seems to be in the region of −10 °C. It should also be noted that the fruit is not cold-hardy.

### 5.2. Other Abiotic Stresses

There are reports of *C. glauca* stands surviving temperatures up to 45 °C with no noticeable effects [42,82,85]. The heat tolerance of other Australian *Citrus* species remains unknown. *C. glauca* is considered somewhat tolerant to salt and boron [57,87,88], which has been exploited in its use as a rootstock [89].

Finally, *C. glauca* has garnered interest as the only known xerophyte from the citrus family [87]. Its physiological adaptations include an extensive root system [90], paraheliotropy [73], a thick cuticle, stomata sunken in deep pits, a palisade parenchyma layer on both the upper and lower surfaces [87], and a lower leaf stomata density [91], which afford a lower net photosynthesis rate and higher water-use-efficiency than other citrus [92]. It can also shed its leaves to reduce water usage and survive extreme drought, with the twigs continuing photosynthesis [42].

### 5.3. Huanglongbing Resistance

Several native Australian Citrus species are well-known for their resistance to huanglongbing (HLB), a bacterial disease caused by ‘*Candidatus liberibacter asiaticus*’ (CLas), which is devastating the global citrus industry. *C. glauca* is fully resistant to CLas [34,93], displaying the highest resistance out of all *Citrus* species and hybrids tested [94]. This is attributed to its high expression levels of the orange1.1g043403m gene [95] and abundant polymorphisms in other pathogen-defence genes [96], including some unique genomic features [97].

*C. australasica* is considered to be moderately resistant to HLB under natural disease challenge conditions [34,93]. Ramadugu et al. [34] noted that *C. australasica* showed an excellent field tolerance, no plants died from HLB exposure, and typical symptoms of HLB were not observed. It was classified in tolerance category 3 (tolerant with some seedling variation). In another study, Alves et al. [93] grafted CLas-infected budwood onto *C. australasica*, finding a partial resistance to infection and also generally low bacterial titer values based on qPCR analyses. Species that were identified as fully resistant in this study included *C. australis* and *C. glauca*.

Other studies have classified *C. australis* as tolerant [34] or resistant to HLB [34,98]. *C. inodora* is considered somewhat resistant but with considerable intra-specific genetic variation [34] and a generally low expression of orange1.1g043403m [95]. It appears that *C. garrawayi* has not been tested for HLB resistance, although it falls in a phylogenetic clade along with other partially resistant *Citrus* species [93]. Similarly, the HLB resistance of *C. gracilis* is completely unknown. Resistance appears to be passed on to hybrids of *C. glauca* [99,100] and *C. australis* [93,99].

Researchers recently isolated a novel class of stable antimicrobial peptides (SAMPs) from *C. australasica* (dubbed MaSAMP), which were the most effective at inhibiting HLB growth out of any *Citrus* SAMPs [101]. Additionally, a MaSAMP foliar spray was able to improve the host immunity against HLB in other *Citrus* species [102]. The genetic basis of the HLB resistance was traced to a range of genes involved in redox control systems and modulating plant defence responses [95,103,104,105,106], including orange1.1g043403m. In another recent study, Zhao and co-workers [107] identified a 14-amino acid peptide (APP3-14) from *C. australasica*, which stabilized the MYC2 immune protein and provided antibacterial (anti-CLas) activity. The application of this protein (delivered by trunk injection) was able to control HLB in infected citrus plants, in both greenhouse and field trials.

### 5.4. Resistance to Psyllids and Other Insects

The Asian Citrus Psyllid (*Diaphorina citri*) is the main vector of HLB; therefore, the host status for *D. citri* plays an important role in determining the HLB susceptibility. *C. glauca* is one of the least-favoured hosts of the *D. citri* [108,109,110], while *C. australasica* [109,111,112,113] and *C. inodora* [108,109] are also less-favoured hosts. *C. australis* is considered a minor host of the *D. citri* [111,112,114], although only adults and no larvae were observed on affected trees [108].

*C. australis* is a host of the Queensland fruit fly (*Bactrocera tryoni*) [115], while *C. australasica* is largely resistant to tephritid fruit flies, including *B. tryoni*, *B. dorsalis*, *Ceratitis capitata*, and *Zeugodacus cucurbitae* [116,117]. Hosts of the Bronze orange bug (*Musgraveia sulciventris*) include *C. australasica* [118], *C. australis* [118], and *C. glauca* [119]. The latter is also a host of the spined citrus bug (*Biprorulus bibax*) [120] and the brown citrus rust mite (*Teolophus australis*) [121], while *C. australasica* hosts the African citrus psyllid (*Trioza erytreae*) [122] and the citrus gall wasp (*Bruchophagus fellis*) [123]. *C. australis* and *C. glauca* are both moderately resistant to the sugar cane root weevil *Diaprepes abbreviatus* [124].

### 5.5. Citrus Canker Resistance

Citrus canker (*Xanthomonas* spp.) is another serious bacterial disease which can affect the leaves and unripe citrus fruit. Asiatic citrus canker (*Xanthomonas citri* pv. *citri*) easily infects *C. garrawayi* [125,126]. *C. glauca* is also considered quite susceptible to *X. citri* pv. *citri* and South American citrus canker (*X. citri* pv. *aurantifolii*) [125,127,128,129], although two contrasting studies have noted little to no citrus canker infestations in the field [130,131].

A study by Licciardello et al. [129] found that the bacterial strain (for both *X. citri* pv. *citri* and *X. citri* pv. *aurantifolii*) affected the infection severity in *C. australis*. This explains why early studies reported that the species was easily infected by Asiatic citrus canker (*X. citri* pv. *citri*) [125,132,133], but others recorded a low disease incidence [131]. In contrast, *C. australasica* is somewhat resistant to all strains of *X. citri* pv. *citri* and *X. citri* pv. *aurantifolii* [125,126,129,132,133].

*C. australis* is also reportedly resistant to bacterial canker (*Xanthomonas campestris* pv. *citri*) [134] and *Phytoplasma aurantiifolia*, the pathogenic bacterium responsible for the ‘Witches broom disease of lime’ [135].

### 5.6. Resistance to Viroids and Viruses

*C. australis* demonstrates a resistance to most citrus viroids, including Citrus exocortis viroid (CEVd), Citrus bent leaf viroid (CVd-I), Hop stunt viroid (CVd-II), Citrus dwarfing viroid (CVd-III), Citrus bark cracking viroid (CVd-IV), and Citrus viroid V (CVd-V) [136,137]. Among these, the best resistance was against CEVd and the poorest was against CVd-V [138]. *C. australasica* is also immune or resistant to CVd-I, CVd-II, CVd-III, and CVd-IV [136]. It should be noted that resistance can be compromised if it is grafted to a more susceptible *Citrus* species, as the viroid can then be spread through the phloem [138].

*C. glauca* is resistant to CEVd [137,138], although sources are divided on its level of resistance to most other citrus viroids. *C. australis* and *C. glauca* are susceptible to the satsuma dwarf virus (SDV) [139]. *C. glauca* appears to be moderately resistant to the Citrus tristeza virus (CTV) [140,141], while *C. australasica* and *C. australis* are both highly susceptible [140,141,142,143], although this varies in *C. australasica* [144]. In these instances, the CTV resistance may be bred by using the *Poncirus* germplasm [145]. Inoculation studies suggest that *C. inodora* is tolerant to the *Citrus psorosis virus* (CPsV) but is not resistant [146] and therefore may demonstrate asymptomatic infections.

### 5.7. Resistance to Fungi and Nematodes

*C. glauca* is considered highly resistant to *Phytophthora parasitica* root rot [51]; *C. australasica* is considered resistant to *Phytophthora citrophthora* (brown rot of citrus) [147] and *Phyllosticta citricarpa* (citrus black spot) [148]. However, *C. australasica* is a host of *Diaporthe citri* (syn. *Phomopsis citri*—the cause of citrus melanose) [149] and a non-symptomatic host of *Elsinoë australis* (sweet orange scab) [150]. *C. australis* is a host of dry rot (*Eremothecium coryli*; syn. *Nematospora coryli*) [151] and citrus scab (*Sphaceloma fawcettii* var. *scabiosa*) [152], amongst others.

*C. australis* is reportedly resistant to nematode infestation [153]; *C. australasica* is also resistant to the burrowing nematode (*Radopholus similis*) [154], but citrus nematode (*Tylenchulus semipenetrans*) infestations can be severe [155].

## 6. Traditional and Contemporary Use

Overall, there is very little published literature on the Indigenous use of Australian citrus. *C. glauca* is one of the few exceptions, with Hatte [156] reporting that tribes of the Isaac Region used *C. glauca* as a food and medicine. Roth [157] recorded its traditional names as *wumbanyi* (Pitta-Pitta tribe; Boulia region) and *kandutal* (Maitakudi or Mayi-Thakurti tribe; Cloncurry region) and noted that the fruit was eaten raw. Finally, the town of Taroom is named after the Waka word ‘*tarum*’, which may mean ‘wild lime’ or ‘pomegranate’ [42].

*C. australasica* is known as *gulalung* in the Bundjalung language. Richmond et al. [158] referenced anecdotal reports that Indigenous tribes used *C. australasica* to prevent illness and as an antiseptic. Furthermore, Packer et al. [159] noted the fruit of two introduced *Citrus* species were used by the Yaegl Aboriginal community (northern NSW) to treat colds (*C. × taitensis* and *C. × sinensis*).

Morrison [64] stated that the *C. australis* fruit was ‘much sought after by both the natives and white settlers’. However, no published information was located on the traditional use of *C. garrawayi* or *C. inodora* by Indigenous tribes. Neither were mentioned by Roth [157] in his list of 240 edible plants used by north Queensland Aboriginal tribes. According to Mabberley [16], the fruit of *C. gracilis* were ‘said to be eaten by Aboriginal people’; however, no reference or clarification was provided and no other scientific literature mentions this.

Early European explorers used *C. inodora* fruit for preserves [50] and *C. glauca* for a ‘gooseberry-fool’ replacement [160] and in jams [161], pickles [73], preserves, and beverages [162]. The latter was featured in cookbooks as early as 1898 [67]. Steeped *C. glauca* flower blossoms were used in jam making [64], while a gum exudate was occasionally eaten [73].

Recent uses of native Australian *Citrus* fruit include in beverages/juices [162,163], sorbets, flavourings [49,164,165], savoury sauces, garnishes [49,166], curries [167], jellies, and tarts [67]. The *C. garrawayi* peel has suggested uses as a candied peel and for grating into spice pastes [168]. Finally, *C. australasica* wood has been used for turning [169] and engraving [162].

## 7. Taxonomy and Hybrids

### 7.1. C. australasica

Its accepted scientific name is *Citrus australasica* F.Muell., with synonyms including *Microcitrus australasica* (F.Muell.) Swingle and *Citrus cataphracta* W.Hill.

Two varieties were recognised by Bailey [170] based on the fruit colour: the type variety *C. australasica* var. *australasica* (black/green/yellow fruit and usually yellow/green pulp) and *C. australasica* var. *sanguinea* F.M. Bailey (with orange/red fruit and usually pink pulp). However, the species is now known to exhibit a wide spectrum of variability [171], making the distinction between the two varieties much less clear than initially thought. Mabberley [16] made no mention of distinct varieties in his taxonomic work returning *C. australasica* to the *Citrus* genus.

Due to its favourable fruit characteristics and disease resistance, *C. australasica* has been cultivated and used in citrus breeding programs for many years. This has led to more than 30 varieties of *C. australasica* stocked in nurseries and grown by Australian *C. australasica* producers [172]. Common cultivars/varieties include ‘Alstonville’, ‘Blunobia Pink Crystal’, ‘Blunobia Red Blush’, ‘Byron Sunrise’, ‘Chartreuse’, ‘Collette’ (from Italy), ‘Durhams Emerald’, ‘Jali Red’, ‘Judy’s Everbearing’, ‘Mia Rose’, ‘Pink Ice’, ‘Pink Pearl’, ‘Rainforest Pearl’, ‘Red Champagne’, ‘Rhyne Red’, ‘UF RedLime’ (in USA), and ‘Yellow Sunshine’.

### 7.2. C. australis

The first European to collect *C. australis* was Allan Cunningham in 1827; this specimen was annotated as *Limonia australis* by Robert Mudie in 1829 and later designated as the lectotype by Mabberley [16]. A fragmentary *Citrus* specimen collected by Ludwig Leichardt in 1845 was described by Planchon as *Citrus australis* [173], while Ferdinand von Mueller redescribed the species as *Citrus planchonii* in 1872.

Its accepted name is now *Citrus australis* (A.Cunn. ex Mudie) Planch., with its synonyms including *Citrus planchonii* F.Muell., *Limonia australis* A.Cunn. ex Mudie, *Limonia australis* A.Cunn. ex G.Do, and *Microcitrus australis* (A.Cunn. ex Mudie) Swingle. It should also be noted that several publications have used *Microcitrus australe*, which is a spelling error and not a valid name.

### 7.3. C. garrawayi

Synonyms of *Citrus garrawayi* F.M.Bailey include *Citrus garrawayae* P.I.Forst., *Microcitrus garrawayi* (F.M.Bailey) Swingle, *Citrus garrowayi* Swingle, *Microcitrus garrawayae* P.I.Forst., and *Microcitrus garrowayi* Swingle. After the species was originally described as *C. garrawayi* by Bailey [59] based on specimens collected by R.W. Garraway from Mount White, Forster [174] noted that the collector was indicated as ‘Mrs’ on various specimen labels. Consequently, Forster changed the specific epithet from the masculine (*garrawayi*) to the feminine form (*garrawayae*). However, the staff of the Queensland Herbarium subsequently confirmed that the species was actually named after Mr. R.W. Garraway (probably Roland Walter Garraway, 1859–1942), thus changing the scientific name back to *C. garrawayi* [55].

A specimen of *Citrus* was collected from Goodenough Island, Papua New Guinea, by Len Brass in 1953 and later misidentified as *C. garrawayi* [174]. Subsequently, *C. garrawayi* was for a time reported as the only *Citrus* species with a native range including both Australia and New Guinea [16]. However, the Papua New Guinean population was later described as a new species: *Citrus wakonai* [58].

### 7.4. C. glauca

The accepted scientific name of Australian desert lime is *Citrus glauca* (Lindl.) Burkill; its synonyms include *Triphasia glauca* Lindl., *Atalantia glauca* (Lindl.) Benth., *Eremocitrus glauca* (Lindl.) Swingle, and *Atalantia glauca* var. *inermis* Bailey. After being incorrectly placed in the *Triphasia* genus (as *T. glauca*) by the botanist John Lindley [175], the species was transferred into *Atalantia*, another near-citrus genera [176]. It was then transferred to its own genus—*Eremocitrus* [73], before finally being returned to the *Citrus* genus by Mabberley [16].

### 7.5. C. gracilis

*C. gracilis* Mabb. was described by Mabberley [16], with the specific name of *Citrus gracilis*, deriving from the ‘graceful aspect of the flowering twigs’ [16]. It should not be confused with *Citrus gracilis* var. *dulcis* Yu. Tanaka, which is a synonym of *C. × aurantium* var. *dulcis*.

### 7.6. C. inodora

Synonyms of *Citrus inodora* F.M.Bailey include *Citrus inodorus* F.M.Bailey, *Citrus maideniana* Domin., *Microcitrus maideniana* (Domin) Swingle, *Microcitrus inodora* (F.M.Bailey) Swingle, and *Pleurocitrus inodora* (F.M.Bailey) Tanaka. *Citrus maideniana* was historically considered a separate species due to distinctive features, including its deeply depressed fruit apex [177], but is now considered a variety of *C. inodora* (var. *maideniana*) [178].

### 7.7. Phylogeny

The phylogenetic relationships of Australian citrus have historically been quite unclear, with different studies providing widely differing results. Wu et al. [11] proposed three ancestral Australian *Citrus* species: *C. australasica*, *C. glauca*, and *C. australis* (Figure 1).

Some taxonomic studies support C. glauca being in its own clade (i.e., the ‘Eremocitrus’ genus) [17,179], while others do not [11,19,180,181,182,183]. However, studies are almost unanimous that C. glauca is most closely related to other native Australian Citrus, compared to international Citrus species [184,185,186].

It is worth noting that the immature fruit of the mangshanyegan (*C. mangshanensis*), a wild citrus species from China, contains globular, stalked juice vesicles very similar to those found in mature *C. australasica* [187]. A recent study also suggested that papeda played an important role in the origin of the Australian finger lime [188]. It has also been noted that *C. gracilis* is superficially similar to the Papuan species *C. wintersii* [16], and this relationship is supported by most phylogenetic studies [19,179,181].

### 7.8. Genomic Characteristics

All of the Australian *Citrus* species tested (*C. australasica*, *C. australis*, *C. glauca*, and *C. inodora*) are diploid, with a chromosome number of 2*n* = 18 chromosomes [189,190]. At least two mitotypes are known to exist in *C. australasica* [191]. Nakandala et al. [97] recently reported the haplotype-resolved genome assemblies of all six native Australian *Citrus* species, with genome sizes ranging from 315 to 391 Mb. These results should provide further clarity into the taxonomic relationships of Australian *Citrus*.

### 7.9. Hybrids

*C. australasica* has been successfully crossed with *C. grandis*, *C. iyo*, *C. macroptera*, *Fortunella margarita*, *Poncirus trifoliata*, *C. inodora* [192], *C. wakonai* [58], *Aegle marmelos* [193], *C. × aurantifolia* [186], *C. × limon*, and *C. madurensis* [194]. However, crosses with *C. glauca* produced no seed [192], while the fruit from the *P. trifoliata × C. australasica* hybrid is inedible [195]. Researchers are also investigating artificial hybridisation techniques [103].

The best-known *C. australasica* hybrid is the Sydney Hybrid (*C. × virgata*), a cross between a male *C. australasica* and female *C. australis* (*C. australasica × C. australis*) [196]. It is resistant to HLB [197] and the pathogenic nematode *Pratylenchus coffeae* [198], making it quite suitable for use as a rootstock. Other common hybrids include the ‘faustrimedin’ (*C. × oliveri*), created by crossing *C. australasica* with calamondin [199]; the ‘faustrime’, from crossing *C. australasica* and *C. × aurantiifolia*; and the ‘faustremon’, from crossing *C. australasica* and *C. × limon*.

*C. glauca* is generally difficult to hybridise [200]; however, hybrids have been reported with *C. wintersii* [201], *C. reticulata* [19], *C. japonica*, and *C. × aurantium* [202]. Hybrids with *C. medica* and *C. × aurantiifolia* died or did not set seed [202]. Its hybrids typically carry an increased boron and salt tolerance [200,203] but are susceptible to *Phytophthora* and herbicides [145]. Researchers are trialling *C. wakonai* as a bridging species to introgress the citrus tristeza virus resistance from *C. trifoliata* into *C. glauca* [204].

The most significant *C. glauca* hybrids include the ‘eremorange’ (*C. glauca* crossed with *C. × aurantium*—sweet orange) and the ‘eremolemon’ (crossed with *C. × limon*). Others are the ‘eremoradia’ (*C. glauca* crossed with *C. × aurantium*—sour orange) [205], ‘citrangeremos’ (eremorange crossed with *C. × insitorum*) [206], the ‘citrangeremo’ (crossed with *C. × insitorum*) [207], the ‘razzlequat’ (crossed with an unknown kumquat) [208], and an unnamed cross with an oval kumquat [209].

## 8. Flavour and Aroma

The flavour of *C. australasica* is described as lime-like, pleasant, and with a distinctive perfume [49]. Smyth et al. [210] conducted the only comprehensive, scientific study of its aroma and flavour, finding that the green-skinned *C. australasica* cultivar showed an aroma of fresh citrus with some cooked notes, while the red-skinned and red-pulp cultivar had an aroma of fresh and cooked citrus, with slight fermented notes. Both cultivars had a ‘citrus’ flavour—tart with some astringency and bitterness [210]. The round pulp vesicles pop in the mouth when eaten, providing a unique texture. Consequently, it has been used as a garnish substitute for caviar, sometimes marketed as ‘caviar citrico’ [211]. The physical structure of the pulp vesicles also has a significant impact on the perceived texture by consumers. One recent study by Nastasi et al. [212] highlighted the variance in the pearl diameter, bursting strength, and strain among pearls from different finger lime varieties

One of the best-tasting species is *C. inodora*, with the Queensland botanist Frederick M. Bailey describing it as ‘of equal flavour with the West Indian Lime’ [213] and as being able to take the place of cultivated lemon, even in its wild state [161]. Other authors agree that the fruit is high-quality [49], describing its flavour as an ‘agreeable acid taste’ [15].

Most authors agree that the flavour of *C. australis* is similar to *C. australasica* but not as good [49,214], being described as ‘slightly bitter with a very tart lime flavour’ [166]. However, Morrison [64] suggested that its quality rivalled ‘the commercial product’ (probably lemons). The fruit produces a sticky exudate which adheres to the fingers and lips, leading Benson [215] to say the skin was full of resinous sap and that the fruit was of little value. Nevertheless, a consumer survey concluded that ‘consumers considered wild lime flavouring in a cheesecake product highly desirable and acceptable’ [166]. It also reported few significant differences in the types and levels of volatile compounds between *C. australis* and Mexican limes, using GC-MS or a sensory analysis.

The *C. glauca* fruit also has a flavour similar to *C. australasica* [216] but with a pleasant, acid (piquant) flavour [42,49,67,160]. The only formal sensory study described its flavour as ‘tart with some astringency and bitterness’, with an aroma of brown lime citrus and fermented notes and some pickled, stewed fruit, and cut grass notes [210]. A juice Brix/acid ratio of ~2.0 provides the optimal flavour [71]. Additionally, its intense flavour means that less quantity is required for flavouring applications [67,85]. Cribb and Cribb [49] indicated that the skin was too aromatic to be eaten, while other authors describe it as virtually tasteless [67].

Sources describe the flavour of *C. garrawayi* as ‘a sharp agreeable acid’ [59] and very similar to lime [50], although no formal sensory study has been performed. There is no scientific literature on the flavour of *C. gracilis*, although Michael Saalfeld anecdotally reported that the pulp had a ‘very strong resinous flavour’ [55].

## 9. Pulp Composition of Commercial and Native Citrus

### 9.1. Nutritional Composition

Like most fruit, citrus contains a high moisture content (>80%) [24], a low ash content (0.1–1.1%) [217], and very little fat (0.1–0.3%) [218]. However, the peel—and particularly the seeds—contains higher levels of unsaturated fatty acids [219,220]. The protein content is also low (0.1–1.3%) [24], with free amino acids comprising most of the nitrogenous constituents [221]. However, citrus fruits are a good source of dietary fibre, containing 1–4.5% *w*/*w* [218]. This includes the soluble dietary fibre pectin and the insoluble dietary fibres cellulose, hemicellulose, and lignin [24]. Meeting recommended dietary fibre intakes can improve colonic health and overall metabolic health and reduce the risk of cardiovascular disease and colorectal carcinoma [222]. Several previous reviews have covered the nutritional composition of commercial citrus species in more detail [24,26,223].

In terms of the native *Citrus* species, the proximate nutritional composition and mineral content of *C. australasica* is reasonably well studied. Like most citrus, the fruit contains high levels of carbohydrates and dietary fibre but little fat or protein (Table 3). The composition of *C. australis* and *C. glauca* is generally similar to *C. australasica*, although the total carbohydrate content of *C. glauca* appears to be lower than other native citrus species. The free amino acid profiles have been reported for *C. australasica* [224], with the most abundant being lysine (26.5 µmol/g), isoleucine (7.6 µmol/g), and arginine (5.6 µmol/g).

No published information could be found on the nutritional composition of *C. gracilis*, *C. inodora* [171,225], or *C. garrawayi* [168], although the moisture content of the latter has been reported at 75.8% [171].

**Table 3:** The nutritional composition and mineral content of the fruit from different samples of *C. australasica*, *C. australis*, and *C. glauca*. Values are reported per 100 g on a dry weight basis, unless otherwise specified.

Species	*C. australasica*						*C. australis*	*C. glauca*	
Variety/Details	Unspecified (DW)	var. *australis* (FW)	var. *sanguinea* (FW)	‘Green’ (DW)	‘Pink’ (DW)	Unspecified (FW)	Unspecified FW)	Frozen/ Fresh (FW)	Freeze-Dried (DW)
Moisture (%)	78.5 ± 1.9	65.5	76.7	-	-	84.2	74.8, 75.4	56.5–78.1, 80.4	-
Energy (kJ)	-	411	-	-	-	176.6	277	198	-
Protein (g)	-	2.5	-	-	-	0.83	2.2	0.1	-
Total fat (g)	-	4.9	1.7	-	-	0.63	BDL	2.7	-
Total saturated fatty acids (g)	-	-	-	-	-		-	1.0	-
Carbohydrates (g)	-	11.7	8.7	-	-	13.7	15.0	4.0	-
Dietary fibre (g)	-	14.0	12.6	-	-		6.7		-
Sugar (g)	-			-	-			4.0	-
Ash (g)	-	0.7	0.7	-	-	0.57	0.8	-	-
Sodium (mg)	11.3 ± 0.1	9	3	11.1	8.7		4	-	2.2
Potassium (mg)	669.7 ± 9.9	290	200	1459.6	1242.6		270	-	1287.8
Magnesium (mg)	57.7 ± 0.5	31	15	139.5	111.1		24	-	94.5
Calcium (mg)	139.0 ± 3.6	50	40	352.7	334.1		46	-	384.2
Iron (mg)	1.24 ± 0.10	0.8	0.6	7.290	3.670		0.5	-	4.740
Zinc (mg)	0.38 ± 0.10	0.3	0.2	0.848	0.780		0.1	-	1.060
Copper (mg)	0.83 ± 0.05	0.4	0.3	0.715	1.31		0.2	-	0.641
Manganese (mg)	0.26 ± 0.02	-	-	0.450	0.400		-	-	0.877
Phosphorus (mg)	87.1 ± 2.4	-	-	166.9	141.7		-	-	127.8
Sulphur (mg)	85.7 ± 7.8	-	-	-	-		-	-	-
Arsenic (mg)	1.87 ± 0.69	-	-	-	-		-	-	-
Aluminium (mg)	0.72 ± 0.17	-	-	0.405	0.644		-	-	3.875
Nickel (µg)	BDL	-	-	34.9	56.3		-	-	48
Molybdenum (µg)	130 ± 230	-	-	10.4	8.3		-	-	7.7
Cadmium (µg)	170 ± 280	-	-	5	4		-	-	5.5
Lead (µg)	140 ± 70	-	-	4	4		-	-	4
Cobalt (µg)	150 ± 60	-	-	2	3		-	-	4
Chromium (µg)	120 ± 80	-	-	-	-		-	-	-
Selenium (µg)	-	-	-	<1	<1		-	-	<1
References	[171,224]	[3]	[3]	[226]	[226]	[227]	[3,171]	[67,171,228]	[226]

BDL = below detection limit; DW = dry weight; FW = fresh weight; and a dash (-) indicates no data available.

### 9.2. Minerals

Citrus fruits such as navel oranges are relatively low in sodium, calcium, magnesium, and phosphorus but are good sources of potassium [218]. Many of these minerals exist in chelated forms with citric acid or other organic acids [229]. The fruit also contains moderate levels of iron, zinc, copper, manganese, and selenium [24]; however, it would not be considered a ‘rich’ source of these trace minerals [230]. Like most species, the citrus mineral composition varies with the soil quality [231] and geographic location [232]. However, several studies have demonstrated that specific *Citrus* rootstocks can provide an increased uptake of certain minerals (e.g., K, Na, P, Cu, Zn, Fe, Mg, and Mn) from the soil [233,234]. Consequently, the mineral content appears to be primarily influenced by environmental factors, but genetic factors do play some role.

Aside from a moderately high arsenic content (in one study) and copper content in *C. australasica*, the mineral composition of most native citrus species is generally without note [226]. Further investigation would be required to confirm if these heavy metals were present due to specific growing conditions/soil types or if it is more characteristic to the species. Most species show a high K:Na ratio—reaching as high as 585 in *C. glauca*, which may help reduce blood pressure [67]. Again, the mineral data has not been reported for *C. garrawayi*, *C. gracilis,* or *C. inodora*.

### 9.3. Sugars and Organic Acids

Most commercial citrus fruit contains 6.4–13.3% carbohydrates by weight [218], with sucrose comprising around 40% of the total sugar content (Table 4), followed by glucose and fructose in approximately equal levels [235]. However, the proportion of individual sugars varies widely between species and even varieties [236]. A few studies have reported low levels of the sugar alcohol inositol in orange and grape juice (0.1–0.4% *w*/*v*) but did not detect any maltose [237]. Despite the relatively high simple saccharide concentrations, clinical studies do not generally find negative health effects associated with citrus or citrus juice intake [238], potentially due to offsetting health benefits from the flavonoids.

Another characteristic feature of citrus fruits is their ‘sour’ taste, primarily due to organic acids. The pulp contains citric, malic, and succinic acid [235], as well as ascorbic acid (see the following section). The peel also contains oxalic, malonic, and quinic acids [243,244]. The organic acid content varies widely in fruit from different citrus cultivars. The organic acid concentration decreases in most varieties (e.g., oranges and grapefruit) as the fruit ripens [245,246]; however, the reverse is true for lemons [247]. Nevertheless, the sugar/acid ratio at maturity remains the main determinant of consumer acceptance. In addition to their important contribution as acid flavouring agents and taste–aroma modulators of other flavours [248], organic acids (particularly citric and malic acid) play an essential role as energy sources for the formation of flavour and aromatic compounds during the ripening process [229,245]. The full complexity of citrate cycling is only recently beginning to be understood [249,250].

Glucose, fructose, and sucrose are the main sugars in *C. australasica*, with some sorbitol also present (Table 5). The total sugar content of the *C. australis* pulp is just over half that found in the Mexican lime, with the main sugars being fructose (4.3 mg/g), glucose (4.2 mg/g), and sucrose (2.4 mg/g) (Table 5). No sugar profiles were found in the literature for *C. glauca*, although its juice ranges from 4 to 12 °Brix [71].

Like most citrus species, citric acid is almost always the dominant acid in *C. australasica* and *C. australis* (Table 6), followed by malic acid and low levels of quinic acid (in *C. australasica*). The exception to this is *C. glauca*, where malic acid is dominant, which is similar to the Palestine sweet lime [243]. Succinic acid has also been reported from *C. australis* [166].

**Table 5:** The sugar content of the native Australian *Citrus* pulp and peel reported in different studies. All values are given as mg/g on a fresh weight basis, unless otherwise specified. Results from *C. × aurantiifolia* are provided as a comparison.

Species	Variety	Growing Location	Sucrose	Glucose	Fructose	Sorbitol	Reference
*C. australasica*	**Pulp**						
	XiangBin	Hainan, China	2.25 ± 0.14	3.34 ± 0.26	4.39 ± 0.31	1.02 ± 0.08	[251]
	LiSiKe	Hainan, China	2.77 ± 0.20	3.15 ± 0.18	4.16 ± 0.28	1.66 ± 0.12	[251]
	Unspecified	Victoria, Australia	ND	1.6–2.6 #	2.4–5.0 #	-	[227]
	‘Red pulp’ (*sanguinea* type) ^1^	Florida, USA	9.65 (mg/mL)	8.48 (mg/mL)	10.10 (mg/mL)	-	[252]
	‘White pulp’ ^1^	Florida, USA	7.54 (mg/mL)	4.37 (mg/mL)	4.22 (mg/mL)	-	[252]
	‘Low-seeded, red pulp, large-leaved’ hybrid ^1^	Florida, USA	2.28 (mg/mL)	0.73 (mg/mL)	2.23 (mg/mL)	-	[252]
	*Sanguinea* type 50–36 cultivar ^1^	Florida, USA	9.90 (mg/mL)	9.85 (mg/mL)	8.46 (mg/mL)	-	[252]
	**Peel**						
	XiangBin	Hainan, China	0.68 ± 0.06	2.08 ± 0.16	2.56 ± 0.22	0.59 ± 0.04	[251]
	LiSiKe	Hainan, China	2.27 ± 0.10	0.71 ± 0.13	0.89 ± 0.19	0.96 ± 0.04	[251]
	**Pulp**						
*C. australis*	Unspecified	Australia	2.4	4.2	4.3	-	[166]
*C. × aurantiifolia*	Unspecified	Australia	5.2	7.4	5.8	-	[166]

^1^ Values obtained from Bikash Adhikari (pers. comm.). # Values provided on a dry weight basis. A dash (-) indicates no data available; ND = not detected.

**Table 6:** The organic acid content of the native Australian *Citrus* pulp and peel reported in different studies. Note that oxalic acid is not included in this table, as it is presented with other anti-nutrients in Table 12. Values are given as mg/g on a fresh weight basis; results from *C. × aurantiifolia* are provided as a comparison.

Species	Variety	Growing Location	Malic Acid	Citric Acid	Quinic Acid	Succinic Acid	Reference
*C. australasica*	**Pulp**						
	XiangBin	Hainan, China	4.08 ± 0.27	73.49 ± 4.10	0.58 ± 0.05	-	[251]
	LiSiKe	Hainan, China	4.80 ± 0.34	71.50 ± 6.47	1.13 ± 0.10		[251]
	‘Red pulp’ (*sanguinea* type)	Florida, USA	5.0 ± 1.1	36.2 ± 3.6	-	-	[252]
	‘White pulp’	Florida, USA	38.0 ± 0.8	37.3 ± 4.2	-	-	[252]
	‘Low-seeded, red pulp, large-leaved’ hybrid	Florida, USA	10.0 ± 3.8	14.6 ± 1.9	-	-	[252]
	*Sanguinea* type 50–36 cultivar	Florida, USA	15.0 ± 3.2	38.8 ± 3.3	-	-	[252]
	‘Green’	QLD, Australia	BDL	46.8 ± 0.5	-	-	[253]
	‘Pink’	QLD, Australia	BDL	58.8 ± 1.7	-	-	[253]
	Unspecified	Australia	-	-	-	-	[171]
	**Peel**						
	XiangBin	Hainan, China	2.23 ± 0.17	20.75 ± 1.57	4.39 ± 0.34	-	[251]
	LiSiKe	Hainan, China	1.35 ± 0.05	8.26 ± 0.22	5.48 ± 0.22	-	[251]
	Unspecified	Valencia, Spain	-	8.11 ± 0.18 ^	-	-	[254]
	**Pulp**						
*C. australis*	Unspecified	Australia	12.0	50.3	-	2.2	[166]
*C. glauca*	Unspecified	QLD, Australia	25.2 ± 0.5	4.61 ± 0.19	-	-	[253]
*C. × aurantiifolia*	Unspecified	Australia	18.6	41.4	-	1.1	[166]

^ Dry weight basis; BDL = below detection limit; and a dash (-) indicates no data available.

### 9.4. Pectin and Other Carbohydrates

*C. australasica* fruits have less pectin than cultivated citrus [49], with Wang et al. [251] reporting 8.3–11.4 mg galacturonic acid equivalents (GTAEs)/g fresh weight of water-soluble pectin in the pulp, which is moderately lower than the peel pectin content.

### 9.5. Vitamins

#### 9.5.1. Commercial Citrus

Perhaps the best-known claim of citrus fruit is their high levels of vitamin C (ascorbic acid), which is among the highest of any common fruit. Levels typically range from 20 to 100 mg/100 g (Table 7), depending on the species and variety. This means that only between 45 and 225 g of citrus would be needed to meet 100% of the daily Recommended Dietary Allowance (RDA) for adults (45 mg) [255]. For comparison, strawberries and papaya typically contain around 60 mg/100 g of vitamin C, while pineapple contains around 48 mg/100 g [218]. Vitamin C levels peak part-way through maturation and decrease thereafter [256,257]. Vitamin C is also slowly lost during postharvest storage [258].

Citrus fruit contains reasonably high levels of several B vitamins (Table 8), most significantly B_1_ (thiamine), B_6_ (pyridoxine), and B_9_ (folate) [26,262]. Citrus juice is considered a particularly good source of folate [263], which plays a crucial role in DNA synthesis and repair and cell division and growth. Values of folate of 16–34 µg/100 mL of orange juice have been reported [264,265], which is comparable to other purported sources of vitamin B_9_ such as broccoli (65 µg/100 g) [218]. Additionally, most folate in citrus is in the stable form of 5-methyl-tetrahydrofolate, meaning there is minimal loss during storage [265]. However, other processing methods may influence B vitamin levels, with Zhu et al. [266] reporting that juicing lemons reduced thiamine but increased riboflavin.

Although pyridoxine is found in the pulp, much higher levels (>10-fold) occur in the peel [262]. A similar trend occurs for most B vitamins in *C. medica* var. *sarcodactylis* [262] and is likely to hold true in other *Citrus* species. Three other B vitamins, namely niacin (B_3_), riboflavin (B_2_) and pantothenic acid (B_5_), are also present in citrus fruit [267], although in lower concentrations (typically 2–4% of their recommended dietary intake [RDI] per serving) [218].

The most abundant group of fat-soluble vitamins in citrus fruit is provitamin A (carotenoids), which is converted into retinol (vitamin A) by oxygenase and reductase enzymes in the small intestine [268]. Total carotenoid levels vary widely between varieties (Table 9), from almost undetectable in white grapefruit cultivars, low/moderate concentrations in oranges, to the highest concentrations in highly pigmented tangerines and grapefruit [269]. The predominant carotenoids are generally lutein/zeaxanthin in oranges, β-cryptoxanthin in tangerines, and β-carotene in red-fleshed fruit (e.g., blood orange, pink pomelo, and red grapefruit) [269]. Citrus fruit contains some of the highest levels of β-cryptoxanthin out of any foods [270].

Carotenoid profiles are under strong genetic control [271,272], with accumulation in the juice sacs induced by blue light wavelengths [273]. Violaxanthin is generally the first to accumulate, followed by β-cryptoxanthin and others [274]. It appears that the carotenoid biosynthesis in the pulp and peel is independent, with Xu et al. [275] suggesting that the exchange of carotenoids between these two tissue types is unlikely to occur.

**Table 9:** The main carotenoids found in common citrus varieties grown in various locations (µg/100 g fresh weight).

Citrus Variety	Origin	α-Carotene	β-Carotene	β-Cryptoxanthin	Lutein + Zeaxanthin	Reference
Grapefruit, pink and red	USA	5	603	12	13	[269]
Grapefruit, red	Spain	-	570 ± 20	10 ± 10	30 ± 30	[276]
Grapefruit, white	USA	8	14	-	-	[269]
Grapefruit, white	Spain	-	ND	ND	ND	[276]
Mandarin (tangerine)	USA	14	71	485	243	[269]
Mandarin	Spain	-	0–240	310–1830	40–90	[276]
Orange, blood	USA	ND	120	69	-	[269]
Orange, blood	Spain	-	40 ± 10	30 ± 10	60 ± 10	[276]
Orange, blood	Spain	-	17	21	Trace	[277]
Orange	USA	16	51	122	187	[269]
Orange	Spain	-	ND	60 ± 20	70 ± 20	[276]
Orange	Spain	-	ND	57	8	[277]
Pumelo, pink	USA	14	320	103	0	[269]
Pumelo	USA	ND	ND	10	-	[269]

ND = not detected; a dash (-) indicates no data available.

Tocopherols (vitamin E) are found in green citrus fruit, but the levels dramatically decrease early in the maturation process [278]. At maturity, the fruit generally contains low tocopherol concentrations in the peel and almost negligible concentrations in the pulp [26]. Nevertheless, tocopherols play an important role in the chilling tolerance of mandarin fruit [279].

One study reported a γ-tocopherol content of 39–83 µg/g DW (dry weight) and an α-tocopherol content of 23–97 µg/g DW in the peel from six Korean citrus species, for a total tocopherol content of 66–131 µg/g DW. The only detectable tocopherol in the pulp was 1 µg/g FW (fresh weight) of α-tocopherol in tangerines [280]. Similarly, the USDA [218] database lists an α-tocopherol content of 0.15 mg/100 g for lemons and 0.18 mg/100 g for citrus. This contrasts with high-vitamin-E plant foods such as peanuts (6.6 mg/100 g) [218]. Additionally, it should be noted that tocopherol contents and profiles are strongly species-specific in *Citrus* [278].

Citrus fruit has negligible concentrations of vitamin K (phylloquinone), at approximately 0.1 µg/100 g [281]. Similarly, no plant-derived vitamin D precursors have been found in citrus [26]. However, the artificial fortification of orange juice products with vitamin D is becoming more common, taking the place of fortified milk in some instances [282,283].

#### 9.5.2. Native Australian Citrus

The vitamin C content of *C. australasica* varies widely depending on the variety (23–115 mg/100 g) but is generally much higher than that found in the Tahitian lime (20 mg/100 g; Table 10). However, the vitamin C content of *C. glauca* is much higher than both orange and *C. australasica* [85], reaching 188 mg/100 g. These high vitamin C contents may be one attractive feature of native Australian citrus for consumers.

Other vitamins found in *C. australasica* fruit include reasonably high levels of vitamin E (higher in the pink cultivar) and moderate concentrations of lutein (provitamin A) (Table 11). Similarly, *C. glauca* is moderately rich in vitamin E, lutein, and folate (vitamin B_9_) [67,228]. The major form of vitamin E in *C. glauca* and both *C. australasica* cultivars was α-tocopherol.

### 9.6. Anti-Nutrients

*C. australasica* does not appear to contain high levels of any common anti-nutrients, such as safrole, cyanogens, oxalic acid, alkaloids, or saponins, although the latter three are present at apparently low to moderate concentrations (Table 12). The alkaloids found in this species do not appear to have been characterised. *C. garrawayi* also contains low levels of oxalic acid and alkaloids but not cyanogens or saponins (Table 12). Screening has shown strong positive results for alkaloids in *C. australis* [171] but low levels of alkaloids and moderate amounts of oxalic acid in *C. glauca*. Additionally, a weak positive result has been reported for cardiac glycosides in this species [286], recommending that a further detailed investigation is required into the anti-nutritional components in *C. glauca*.

**Table 12:** Anti-nutrients found in different native *Citrus* species. Multiple entries in the same cell are from different studies. Where applicable, values are reported as g per 100 g on a dry weight basis.

Analyte	*C. australasica*	*C. australis*	*C. garrawayi*	*C. glauca*
Oxalic acid	0.11 ± 0.04, 0.8, 2.0	0.09	0.13	<0.8, 1.04 ± 0.10, 1.7
Cyanogens	BDL (<0.1)	BDL (<0.1)	BDL (<0.1)	BDL (<0.1)
Alkaloids	+ve (>0.04)	Strongly +ve	Slight +ve (>0.04)	Sometimes +ve (>0.04)
Saponins	+ve (in 1 of 4 samples)	BDL	BDL	+ve, BDL
Safrole	BDL	-	-	-
Cardiac glycosides	-	-	-	Slight +ve
References	[171,253]	[171]	[171]	[171,253,286]

BDL = below detection limit; a dash (-) indicates no data available. Alkaloid, saponin, and cardiac glycoside data comes from qualitative screening assays. ‘+ve’ means a positive response to the assay, ‘strongly’ means a strong response (as reported by the authors), ‘slight’ means a small response, and ‘sometimes’ means multiple samples were tested but with contrasting results (some positive and some negative).

### 9.7. Phytochemical Composition

#### 9.7.1. Commercial Citrus

Citrus fruit contains secondary metabolites, dominated by polyphenols and particularly flavonoids [267]. Polyphenols are a broad class of compounds, defined by the presence of several hydroxyl groups attached to one or more aromatic rings. Out of all the secondary metabolites produced by plants, polyphenols have attracted significant interest due to their near-ubiquitous antioxidant activity [287], leading to other beneficial biological activities [288,289]. Common subclasses of polyphenols include phenolic acids, stilbenes, lignans, and flavonoids [290].

Common phenolic acids found in citrus fruit include gallic acid, *trans*-ferulic acid, *p*-coumaric acid, sinapic acid, caffeic acid, and *trans*-cinnamic acid [267,291]. The phenolic acid concentrations are usually higher in the peel compared to the pulp [291].

**Figure 14:**
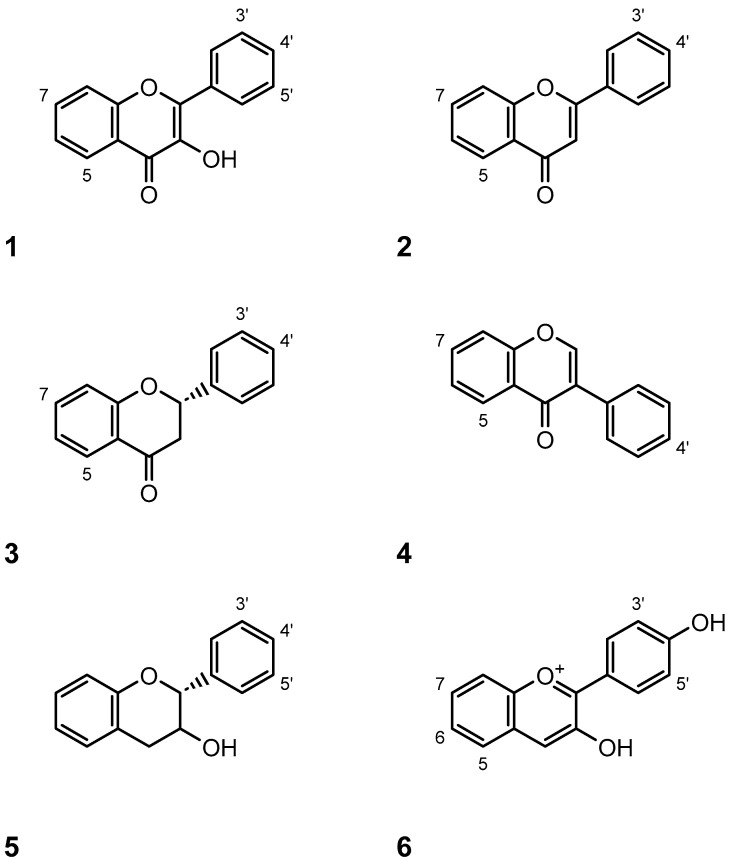
The basic molecular structures of the six flavonoid classes: (**1**) flavonols, (**2**) flavones, (**3**) flavanones, (**4**) isoflavones, (**5**) flavan-3-ols (catechins), and (**6**) anthocyanidins. The numbered carbons indicate where common substituents (OH or OCH_3_) occur. Figure by Joel Johnson, following Pietta [296]; reproduced under the Creative Commons 4.0 licence (https://doi.org/10.6084/m9.figshare.27001750).

Another group of polyphenols are the flavonoids, which have a characteristic 15-carbon backbone structure (see Figure 14), often modified by the addition of *O*-methylated, hydroxyl, or glycosyl groups. As with phenolic acids, flavonoids are generally found in higher concentrations in the peel compared to the pulp [292]. Flavonoids are further divided into six classes (Figure 14), four of which occur widely in citrus: flavones, flavanones, flavonols, and flavans. Anthocyanins also occur in the red pulp of the blood orange [293,294], with cyanidin 3-glucoside and cyanidin 3-(6″-malonylglucoside) being the predominant types [295].

The main flavonoid compounds found in citrus include hesperidin (a flavanone rutinoside) and naringin (a flavanone neohesperidoside with a bitter taste). For more detail on *Citrus* flavonoids and their properties, the interested reader is referred to a review by Tripoli et al. [297]. Hesperidin has garnered particular interest, as it shows anti-obesity [298], neuroprotective [299], and anticancer activity [300]. Furthermore, some studies suggest that hesperidin levels increase as the fruit matures [301], in contrast to most other flavonoids which decrease during the maturation process [302,303]. Naringin and its corresponding aglycone, the flavanone naringenin, have also attracted interest as they appear to possess similar bioactive properties to hesperidin [304,305]. However, the naringin content decreases during maturation [306].

**Figure 15:**
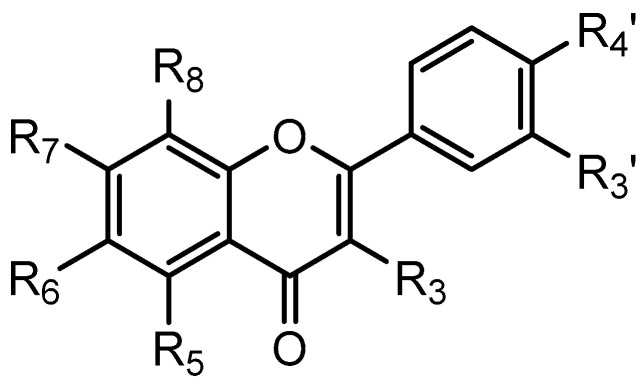
The general structure of polymethoxyflavones. The R_3_ to R_8_ and R_3′_ to R_4′_ substituents may be H, OH, or OMe, with the total number of OMe groups being between 2 and 7. Figure by Joel Johnson, following Li et al. [314]; reproduced under the Creative Commons 4.0 licence (https://doi.org/10.6084/m9.figshare.27001924).

Another group of flavonoids—which are unique to the *Citrus* genus—are the polymethoxyflavones [307]: flavones bearing two to seven methoxy groups from their basic benzo-γ-pyrone skeleton (Figure 15) [308]. Common polymethoxyflavones include nobiletin, tangeretin, and sinensetin [308]. These are believed to be responsible for many of the unique health benefits of citrus fruit, such as their anti-inflammatory activity [309,310] and anti-obesity effects [311,312]. Polymethoxyflavones may also inhibit the proliferation of cancer cells [313].

Some citrus cultivars produce high concentrations of lycopene, a non-provitamin A carotenoid that imparts the red colour of tomatoes and watermelons [275]. Consequently, lycopene-producing cultivars usually have a red pulp, such as the red pumelo and Ruby Red grapefruit. In contrast, the bright red colour of blood oranges is produced by anthocyanins, a class of flavonoids [294].

**Figure 16:**
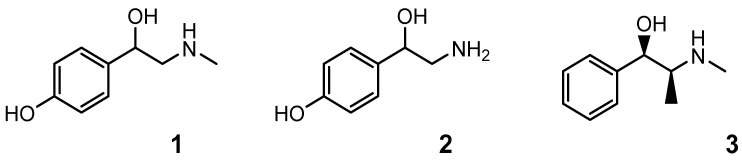
The structure of *p*-synephrine (**1**), octopamine (**2**), and ephedrine (**3**), a known stimulant. Figure by Joel Johnson; reproduced under the Creative Commons 4.0 licence (https://doi.org/10.6084/m9.figshare.27001936). Drawn in ChemDraw 23.1.2 by Joel Johnson.

Another unique class of compounds found in some citrus varieties is the synephrines, which are phenethylamine alkaloid derivatives, and include *p*-synephrine and octopamine. Bitter orange (*C. × aurantium*) contains the highest synephrine levels, with *p*-synephrine (Figure 16, structure 1) comprising around 90% of the synephrine content in this species [315]. However, synephrines are found in most citrus fruit at concentrations of 0.04–0.20% *w*/*w* [316], even reaching 73–158 mg L^−1^ in the juice from *C. × unshiu* mandarins [317]. Concerns have been raised over the safety of synephrines due to their structural similarity to the stimulant ephedrine (Figure 16, structure 3), although synephrines do not appear to have significant stimulant effects at the concentrations typically found in citrus fruit [315].

#### 9.7.2. Native Australian Citrus

Qi et al. [318] recently published a detailed review including the nutritional value and phytochemical constituents found in *C. australasica* and their potential health benefits; hence, only an overview is provided here. Compared to most other native fruit, *C. australasica* appears to have relatively low levels of phenolics [4,253]. The level of total phenolics in the pulp does also not appear to vary greatly (i.e., staying well within the same order of magnitude) between different coloured *C. australasica* varieties [4,252,253,284].

Wang et al. [251] used LC-MS to tentatively identify 31 phenolic compounds from *C. australasica*, including a secoiridoid derivative and a neolignan glycoside from two *C. australasica* cultivars; the most abundant phenolics in the pulp were quercetin 3-rutinoside-7-glucoside and chrysoeriol 7-*O*-rutinoside. However, no polymethoxyflavonoids were identified. Using HPLC-QT of mass spectrometry, Aznar et al. [254] identified 15 compounds (4 tentatively) from *C. australasica*, including 7 not previously reported from this species (4-vinylphenol, hyperoside, *o*-coumaric acid, ononin-*O*-acetate, pyrogallol, deacetylnomilinic acid, and limonin). The most abundant phenolics were naringin, didymin, and *o*-coumaric acid. Cioni et al. [319] identified 26 compounds (23 tentatively) in the *C. australasica* peel and pulp using UHPLC-DAD-HR-Orbitrap/ESI-MS, including 5 anthocyanins; however, the only liminoid tentatively identified was limonexic acid. Finally, Cáceres-Vélez et al. [320] identified 32 compounds (24 tentatively) using HPLC-ESI-QTOF-MS/MS, predominantly including flavonoids as well as some phenolic acids and glycosides.

**Figure 17:**
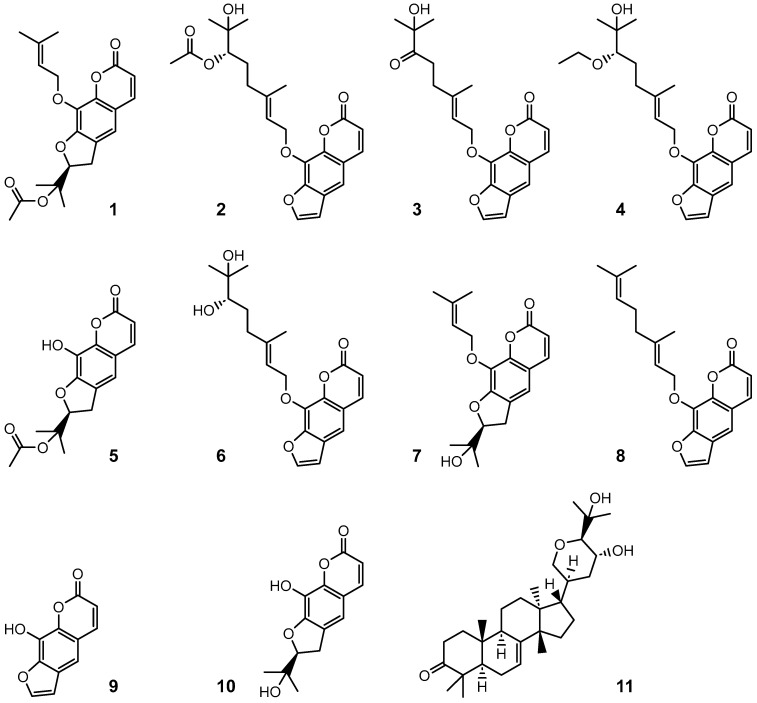
The 11 compounds identified from *Citrus garrawayi* fruit by Raju et al. [321] via anti-inflammatory activity-guided fractionation. The compounds are (**1**–**5**) Garracoumarins A-E, (**6**) (*S*, *E*, *E*)-8-(6,7-dihydroxyl-3,7-dimethyloct-2-en-1-yl)oxyl-psoralen, (**7**) (+)-8-(3-methylbut-2-enyloxyl)-marmersin, (**8**) bergamottin (5-geranyloxypsoralen), (**9**) xanthotoxol (8-hydroxylpsoralen), (**10**) rutaretin, and (**11**) bourjotinolone A. Structures were drawn by the authors in ChemDraw, following Raju et al. [321]. The figure is by Joel Johnson, reproduced under the Creative Commons 4.0 licence (https://doi.org/10.6084/m9.figshare.27002239).

Raju et al. [321] used anti-inflammatory activity-guided fractionation to identify eleven compounds from *C. garrawayi* fruit extracts: five new compounds (named garracoumarins A-E), five known isoprenylated furanocoumarins, and the known sterol bourjotinolone A (Figure 17). Many of the known compounds had been previously found in other *Citrus* species [322,323]. Garracoumarins C and E showed the strongest inhibition of nitric oxide (NO)—although not as high as bourjotinolone A—while garracoumarins C and D provided the strongest inhibition of the TNF-α production out of all compounds isolated [321].

Compared to other native fruits, *C. glauca* has a moderately high antioxidant capacity [324,325], mainly attributed to its hydrophilic fraction [228]—in contrast to *C. australasica* [251,252,284]. Flavonoids reported from the fruit pulp/juice include hesperidin (0.9 mg/g FW), narirutin (0.1 mg/g FW), and trace levels of narirutin-4′-glucoside and eriocitrin [326,327], while the coumarins 2′,3′-dihydroxydihydrosuberosin and 7-hydroxycoumarin have been identified from a *C. glauca* hybrid [328]. Bashir [329] recently reported on 108 compounds (more than half of which were identified) from *C. glauca* fruit, including flavonoids, catechin, procyanidins, and phenolics. Of these, 21 were identified as being antioxidant-active.

Finally, *C. inodora* is notable for having the highest juice flavone/flavonol concentration out of any *Citrus* species studied, highlighting the need for characterising its phenolic (and particularly flavone/flavonol) content [326]. Previous work has only reported the flavanone naringin-6″-malonate (0.2 mg/g FW) and low levels of coumarins (0.05 mg/g) from this species [326].

### 9.8. Volatiles

A number of studies have reported on the juice volatile profiles of different *C. australasica* cultivars. As shown in Table 13, d-limonene tends to be the dominant compound, although it is generally found at lower concentrations than in the peel. One study on Californian-grown fruit used chiral GC to confirm that 99.1% of d-limonene in the juice was the (*R*)-(+) enantiomer [330], matching results from the peel. The major aroma-active compounds identified in *C. australasica* juice are linalool, myrcene, d-limonene, isomenthone, and citronellol [330].

Shaw et al. [38] conducted an investigation into the juice volatiles of *C. inodora*, using Florida-grown fruit. In total, 53 volatile compounds were identified, with the major components being d-limonene (68.5% of GC area), ethanol (14.6%), acetaldehyde (9.4%), myrcene (1.44%), and hexanal (0.63%).

### 9.9. Bioactive Properties

The abundant bioactive secondary metabolites and vitamins imbue citrus fruit with a range of beneficial health effects [336]. Clinical studies demonstrate that consuming oranges, orange juice, or orange extracts can reduce the body weight in overweight populations [337,338], decrease plasma lipids and triglycerides [339], reduce inflammation [263], and reduce the overall cancer risk [340]. Much of the anti-inflammatory activity has been attributed to the flavonoids naringin, hesperitin, and hesperidin [301,341]. Depending on the flavanone bioavailability and gut microbiota, the citrus consumption may also reduce the risk of developing type 2 diabetes [342]. Consequently, this supports the importance of citrus fruit as a nutritious and health-promoting fruit which can comprise an important part of the human diet. This is particularly important given the increasing global burden of diabetes [343].

*C. australasica* extracts show dose-dependent in vitro anti-inflammatory activity, mediated through inhibiting the release of nitric oxide (NO) and the proinflammatory cytokines IL-1β, IL-6, and TNFα [251]. Gene expression studies found that this was primarily mediated through the *STAT3* pathway.

Similarly, garracoumarins C and D isolated from *C. garrawayi* showed a strong inhibition of the TNF-α production in vitro, while bourjotinolone A inhibited the NO production [322], this may provide anti-inflammatory activity.

*C. australasica* extracts also contain alpha hydroxy acids which can activate the Transient Receptor Potential Vanilloid-3 (TRPV3) of keratinocytes, causing skin desquamation and renewal [344]. An extract of the alpha hydroxy acids (AHAs) is marketed as Lime Pearl™ by Lucas Meyer Cosmetics (Knockrow, NSW); *C. glauca* extract has also been used in other cosmetic products [345].

*C. glauca* fruit extracts showed a moderate inhibitory activity against *Shewanella putrefaciens* [286] and *Streptococcus pyogenes* [346] but not *Bacillus anthracis* [347]. The extracts could also significantly inhibit the HeLa and CaCo-2 cancer cell growth [325]. Similarly, Forbes-Smith and Paton [166] found that *C. australis* juice displayed moderate antimicrobial activity against several bacteria species, particularly *Erwinia carotovora* and *Bacillus cereus*.

It should be noted that recent in vivo toxicological testing suggested *C. australasica* extracts may adversely affect zebrafish embryos at very high concentrations (>480 mg/L) [320,348], so more work on the fruit’s safety may be required. *C. glauca* extracts displayed no toxicity in an *Artemia franciscana* nauplii assay [286].

The Expert Panel for Cosmetic Ingredient Safety has deemed the *C. glauca* fruit (pulp) extract (CAS number 1174331-62-4) to be safe for cosmetic use at low concentrations (0.003–0.005%) [349], with reported uses as an humectant and antistatic/hair conditioning agent.

## 10. Peel Composition

### 10.1. Vitamins

The vitamin C content of the *C. australasica* peel (21–49 mg/100 g FW) is generally comparable to that of the Tahitian lime peel (37 mg/100 g FW), as shown in Table 14 No other studies appear to have investigated the vitamin contents of the peel.

### 10.2. Pectin and Anti-Nutrients

Wang et al. [251] reported a water-soluble pectin content of 14.9–18.7 mg galacturonic acid equivalents (GTAEs)/g FW in the peel, which is higher than the pulp but generally lower than most other citrus species. Screening tests have shown strong positive results for alkaloids in *C. australis* peels [171].

### 10.3. Phytochemical Composition

The antioxidant activity of *C. australasica* is typically higher in the peel than in the pulp [251,252,284] and can be mainly attributed to the lipophilic constituents [226,254]. The *C. australis* peel also shows a moderate antioxidant activity using the β-carotene bleaching agar diffusion test [166].

The *C. glauca* peel contains naringin glucoside, rutin [327], hesperidin (1.7–1.8 mg/g FW), naringin-4’-glucoside (0.3–0.5 mg/g FW), naringin (0.1 mg/g FW), and narirutin (0.2 mg/g FW) [326].

The only study found on the phytochemical content of *C. inodora* fruit was conducted by Kanes et al. [327] and was later expanded by Berhow et al. [326]. These authors tentatively reported 33 phenolic peaks in the flavedo, noting that it contained the highest concentration of flavones/flavonols (8.75 mg/g) out of all citrus species tested. However, the only flavanone identified was naringin-6″-malonate (closed form; 0.4 mg/g FW), while coumarins were observed at very low concentrations (0.02 mg/g).

### 10.4. Volatiles

#### 10.4.1. Commercial Citrus Species

The volatile compounds found in citrus fruit (primarily in the peel) are responsible for their unique ‘citrus’ aroma and may also contribute to their characteristic flavour. Terpenoids are the most common volatiles in citrus essential oil [350] and are also the most abundant free volatiles found in citrus juice [351]. Across a number of *Citrus* species, the monoterpene d-limonene is often reported as the most abundant individual volatile compound, while other major volatiles include β-myrcene, α-pinene, β-pinene, sabinene, and γ-terpinene [350].

Closely related *Citrus* species tend to display similar volatile profiles [350,351]. In general, the peel of *C. reticulata* (mandarin), *C. × sinensis* (sweet orange), *C. maxima* (pummelo), *C. × aurantium* (bitter orange), and *C. × paradisi* (grapefruit) contains a high number of non-terpenoid esters and aldehydes [350]. On the other hand, the peel volatile profile of less commonly consumed citrus, such as *C. medica* (citron), *C. × bergamia* (bergamot orange), *C. × junos* (yuzu), and *C. × aurantiifolia* (lime), is dominated by monoterpenes and sesquiterpenes [350]. Lemon (*C. × limon*) contains some unique sulphur-containing monoterpenoids and non-terpenoid esters not found in most other species [350].

#### 10.4.2. Native Australian Citrus Species

**Figure 18:**
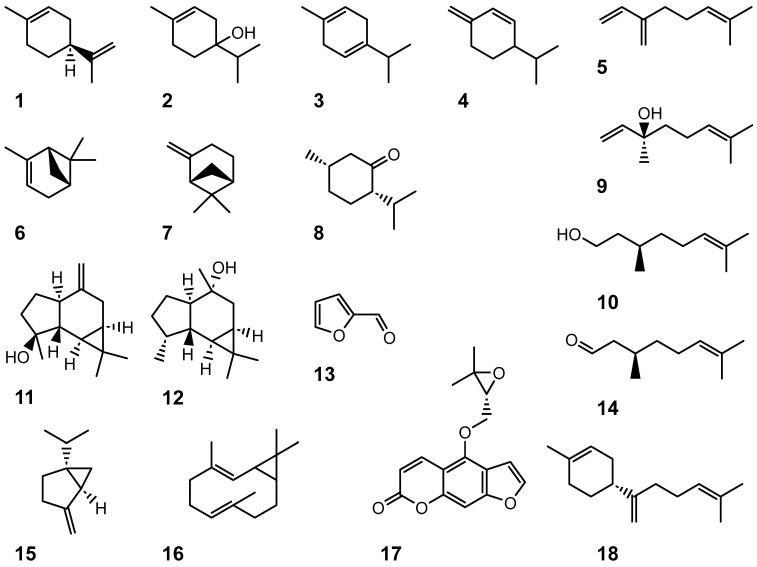
Some of the predominant volatile compounds reported from *C. australasica* and its hybrids. (**1**) (*R*)-(+)-d-limonene, (**2**) terpinen-4-ol, (**3**) γ-terpinene, (**4**) β-phellandrene, (**5**) β-myrcene, (**6**) (1*R*,5*R*)-(+)-α-pinene, (**7**) (1*S*,5*S*)-(−)-β-pinene, (**8**) (1*S*,4*S*)-(−)-isomenthone, (**9**) (*R*)-(−)-linalool, (**10**) (*R*)-(+)-β-citronellol, (**11**) spathulenol, (**12**) globulol, (**13**) furfural, (**14**) (*R*)-(+)-citronellal, (**15**) (*S*)-(−)-sabinene, (**16**) bicyclogermacrene, (**17**) oxypeucedanin, (**18**) β-bisabolene. Where applicable, the stereochemistry of chiral compounds follows the major stereochemistries found by Delort et al. [352] and Harada [330] in *C. australasica*. The figure is by Joel Johnson, reproduced under the Creative Commons 4.0 licence (https://doi.org/10.6084/m9.figshare.28570373).

Most work on the chemical composition of *C. australasica* has focused on the peel volatile profile. Its unique features include the dominance of d-limonene and having isomenthone as a major component (7.5%) [352]. The structures of the major volatile compounds identified from the *C. australasica* peel are shown in Figure 18. The large amount of genetic diversity in this species [70,353] leads to a diverse array of volatile chemotypes, which are shown in Table 15. Apart from the detailed information in this table, only the key volatile studies are mentioned in the remainder of this section.

The pioneering work by Delort and Jaquier [354] reported 195 volatile compounds from *C. australasica*, followed by identifying 6 novel terpenyl esters [352]. Additionally, almost all (96.7–99.4%) d-limonene present was the (*R*)-(+)-d-limonene enantiomer, which is the most common limonene enantiomer present in the *Citrus* genus [355,356]. Another study using chiral GC on fruit sourced from California reported that 99.0% of the d-limonene present in the peel was the (*R*)-(+) enantiomer [330]. The same study reported the major enantiomers of citronellol, isomenthone, β-pinene, α-pinene, linalool, and α-terpineol as (*S*)-(−)-citronellol (>99%), (1*S*,4*S*)-(−)-isomenthone (>99%), (1*S*,5*S*)-(−)-β-pinene (>99%), (1*R*,5*R*)-(+)-α-pinene (91.0–95.4%), (*R*)-(−)-linalool (76.9–79.9%), and (*S*)-(−)-α-terpineol (58.2%). Cucinotta et al. [357] reported the major enantiomers as follows: (−)-α-thujene (92.3–95.7%), (+)-α-pinene (78.4–84.5%), (−)-camphene (56.3–61.1%), (+)-sabinene (87.4–91.1%), (+)-β-pinene (88.9–94.2%), (+)-α-phellandrene (99.3–99.4%), (+)-limonene (88.1–89.3%), (+)-β-phellandrene (96.8–98.9%), (−)-linalool (72.9–81.6%), (−)-citronellal (99.7–99.8%), (+)-α-terpineol (71.2–75.8%), and (−)-citronellol (78.9–79.6%). The stereochemistry of terpinen-4-ol was almost evenly split, with the proportion of (+)-terpinen-4-ol ranging from 43.2 to 56.4%. This differs from the previous study [330] in the stereochemistry of β-pinene and α-terpineol. Additionally, Delort et al. [352] reported the major stereochemistry (95.8–97.2%) of citronellol as (*R*)-(+)-citronellol, highlighting that further work may be required to clarify the true enantiomeric forms of some compounds from this species.

Later studies expanded the number of volatile compounds tentatively identified to 447 [224] and compared a larger number of *C. australasica* varieties (5), albeit without reporting any novel compounds [284]. Finally, D’Auria and Racioppi [331] were the first to use headspace solid phase micro-extraction (HS-SPME) with GC-MS, allowing for a volatile analysis with no sample extraction or preparation required. Again, all of these volatile compounds had been previously reported.

Only one study has reported on the major aroma-active compounds from *C. australasica*, i.e., those present at a concentration higher than their perception threshold. Using an aroma extract dilution assay, the major aroma compounds of the peel were reported to be linalool (flavour dilution [FD] factor of 12), citronellol (FD factor 11), citronellal (FD factor 9), and (*Z*)-9-dodecen-12-olide (FD factor 8) [330].

Forbes-Smith and Paton [166] and Craske et al. [358] used GC-MS to compare the volatile compounds in *C. australis* and Mexican lime (*C. × aurantiifolia*) peels, reporting few overall differences. The major volatiles in the *C. australis* peel were d-limonene (35–38%), β-pinene (13–14%), and γ-terpinene (11–12%) (Table 16)—which is again similar to *C. australasica* but not having isomenthone as a major component. Compared to *C. × aurantiifolia*, the major differences were more d-limonene and less γ-terpinene and neryl acetate in *C. australis*. Additionally, the aroma was reportedly very similar to *C. × aurantiifolia* [358].

The only investigation into the peel volatiles of *C. inodora* was conducted by Shaw et al. [38], using Florida-grown fruit. Twenty volatile compounds were identified, principally d-limonene (68.5% of GC area), ethanol (14.6%), acetaldehyde (9.4%), myrcene (1.44%), and hexanal (0.63%). Several of the trace volatiles are not commonly found in most *Citrus* species, being tentatively identified as 3-methyl-2,5-furandione, elemol, linalyl-3-methybutanoate, 4,9-dimethoxy-psoralen, and 7-[(3,7-dimethyl-2,6-octadienyl)oxy]-coumarin [38].

The peel volatile profiles of *C. garrawayi*, *C. glauca*, and *C. gracilis* do not appear to have been reported to date.

**Table 15:** The main chemotypes reported for the peel of various *C. australasica* cultivars, along with *Citrus australis*. The first-, second-, and third-most abundant volatile compounds are provided for each variety. Values are given as percentages of the total volatile composition. The chemotype of Tahitian lime is reported at the bottom of the table (bold entry) for reference.

Variety	Most Abundant Compound	Second-Most Abundant Compound	Third-Most Abundant Compound	Reference
Alstonville	d-limonene (61.7%)	sabinene (20.6%)	oxypeucedanin (5.7%)	[352]
Chartreuse	d-limonene (61.4%)	β-citronellol (6.7%)	citronellal (6.5%)	[284]
Collette ^#^	d-limonene (42.4%)	γ-terpinene (14.2%)	terpinen-4-ol (8.4%)	[319]
Durham’s Emerald	d-limonene (66.3%)	citronellal (9.3%)	citronellol (5.2%)	[352]
Durham’s Emerald	d-limonene (73.8%)	β-citronellol (5.8%)	citronellal (5.6%)	[284]
Hybrid ‘P1f2-10’	d-limonene (83.7%)	bicyclogermacrene (2.9%)	γ-terpinene (2.5%)	[284]
Judy’s Everbearing	d-limonene (64.4%)	citronellal (9.0%)	isomenthone (7.3%)	[352]
Pink Ice ^#^	d-limonene (37.7%)	sabinene (33.3%)	α-pinene (5.6%)	[331]
Pink Ice ^#^	Terpinen-4-ol (38.3%)	limonene (26.5%)	γ-terpinene (7.3%)	[319]
Pink Pearl ^#^	d-limonene (63.2%)	sabinene (9.5%)	bicyclogermacrene (7.2%)	[332]
‘Red’ ^#^	d-limonene (73.6%)	bicyclogermacrene (6.9%)	β-bisabolene (2.0%)	[319]
Red Champagne	d-limonene (87.5%)	bicyclogermacrene (4.1%)	β-myrcene (2.2%)	[284]
Rhyne Red	d-limonene (65.0%)	γ-terpinene (16.8%)	citronellal (2.4%)	[284]
Unspecified	d-limonene (6.9%) ^1^	α-pinene (5.5%)	furfural (4.3%)	[224]
Unspecified	d-limonene (51.6%)	isomenthone (9.7%)	linalool (7.5%)	[330]
Unspecified	d-limonene (73.5%)	isomenthone (7.5%)	citronellal (2.6%)	[354]
Unspecified ^#^	d-limonene (51.1%)	sabinene (19.6%)	β-pinene (7.9%)	[359]
Unspecified ^#^	d-limonene (24.5–38.9%)	citronellal (7.2–23.7%) ^2^	β-phellandrene (13.2–18.2%) ^2^	[357]
Unspecified ^#^	d-limonene (62.6%)	β-pinene (32.4%)	α-pinene (1.5%)	[333]
var. *sanguinea* ^#^	bicyclogermacrene (25.9%)	α-pinene (10.2%)	spathulenol (9.8%)	[360]
var. *sanguinea* ^#^	d-limonene (65.7%)	γ-terpinene (8.8%)	bicyclogermacrene (7.0%)	[332]
var. *sanguinea* ^#^	d-limonene (48.2%)	sabinene (37.2%)	α-pinene (4.3%)	[331]
Yellow Sunshine ^#^	d-limonene (40.0%)	bicyclogermacrene (39.8%)	globulol (2.9%)	[319]
*C. australasica* × *C. inodora* (‘Minnie finger lime’)	d-limonene (82.4%)	β-myrcene (6.5%)	α-pinene (2.1%)	[361]
Faustrime (*C. australasica × C. × aurantiifolia*) ^#^	d-limonene (43.2%)	citronellal (16.3%)	γ-terpinene (11.8%)	[362]
Faustrime (*C. australasica × C. × aurantiifolia*) ^#^	citronellal (22.2%)	β-phellandrene (17.7%)	limonene (17.2%)	[363]
Faustrime (*C. australasica × C. × aurantiifolia*) ^#^	d-limonene (31.5%)	γ-terpinene (11.6%)	citronellal (9.4%)	[319]
Faustrime (*C. australasica × C. × aurantiifolia*) ^#^	d-limonene (29.3%)	β-phellandrene (21.1%)	γ-terpinene (9.5%)	[332]
Faustrime (*C. australasica × C. × aurantiifolia*) ^#^	d-limonene (27.8%)	citronellal (10.5%)	γ-terpinene (10.0%)	[331]
Faustrime (*C. australasica × C. × aurantiifolia*) ^#^	citronellal (23.5%)	d-limonene (13.0%)	citronellol (10.7%)	[334]
***Citrus australis***				
*Citrus australis*	d-limonene (38.2%)	β-pinene (14.3%)	γ-terpinene (12.2%)	[166]
*Citrus australis*	d-limonene (35.1%)	β-pinene (13.1%)	γ-terpinene (11.2%)	[358]
**Commercial Tahitian Lime** **(*Citrus × latifolia*)**	**d-limonene (40.3%)**	**γ-terpinene (13.4%)**	**β-pinene (10.9%)**	[284]

^#^ The fruit investigated in this study were grown outside of Australia. Notes: Faustrime = *C. australasica* × *Fortunella* sp. × *C.* × *aurantiifolia*; var. *sanguinea* = *Citrus australasica* var. *sanguinea*. ^1^ Data obtained from Vuanghao Lim (pers. comm.). ^2^ In one season studied, citronellal was the 3rd most abundant compound and β-phellandrene was the 2nd. Shaded rows indicate hybrids or citrus species which are not native Australian citrus species.

**Table 16:** Volatile constituents found in the peel of *Citrus australis*, in comparison to the Mexican lime (*Citrus × aurantiifolia; shown in grey shading*). Note that compounds unique to a particular species were only found at trace concentrations (<0.5%).

Compound.	*C. australis* (%)	*C. australis* (%)	*C. × aurantiifolia* (%)	Odour Description ^1^
α-thujene	<0.5	0.2	<0.5	Sweet, rose, spicy
α-pinene	1.41	1.3	1.45	Lemon
sabinene	2.45	2.2	1.41	Off-lemon, faint lemon
β-pinene	14.28	13.1	8.4	Lime
β-myrcene	0.99	1.0	0.97	-
α-terpinene	<0.5	0.1	<0.5	Rose, lemon
*p*-cymene	BDL	0.1	BDL	-
d-limonene	38.22	35.1	31.65	Grassy, leafy
1,8-cineole	BDL	BDL	<0.5	Menthol
(*E*)-β-ocimene	<0.5	0.2	BDL	-
γ-terpinene	12.17	11.2	20.4	Menthol
terpinolene	<0.5	0.4	0.87	Plastic
linalool	>0.5	0.3	<0.5	Lime
isoborneol	BDL	BDL	<0.5	Floral
α-terpineol	<0.5	0.7	<0.5	Lemon orange, rose, lemongrass
decanal	<0.5	0.8	<0.5	-
neral	4.91	4.5	4.02	-
geranial	7.93	7.3	6.31	Faint orange
δ-elemene	1.25	1.2	<0.5	Faint lemongrass
neryl acetate	<0.5	0.1	2.3	-
geranyl acetate	<0.5	0.4	<0.5	Grassy
β-elemene	<0.5	0.7	<0.5	-
Unidentified	<0.5	0.3	BDL	-
(*E*)-caryophyllene	<0.5	1.0	<0.5	-
γ-elemene	<0.5	0.2	<0.5	-
α-*trans* bergamotene	1.76	1.6	1.84	-
germacrene D	<0.5	0.5	<0.5	-
α-garnesene (*E,E*)	3.57	3.3	BDL	-
(*Z*)-α-bisabolene	BDL	0.1	<0.5	Faint lemon
unidentified	BDL	BDL	<0.5	-
β-bisabolene	3.04	2.8	2.99	-
germacrene B	<0.5	0.9	<0.5	-
7-methoxy coumarin	<0.5	1.1	3.48	-
unidentified	BDL	BDL	<0.5	-
5,7-dimethoxy coumarin	3.66	3.4	7.08	-
iso-bergaptene	<0.5	0.2	3.04	-
bergaptene	<0.5	0.4	<0.5	-
isopimpinellin	3.78	3.5	1.94	-
Reference	[166]	[358]	[166]	[166]

^1^ From GC-olfactory analysis; BDL = below detection limit; a dash (-) indicates no data available.

### 10.5. Bioactive Components

The peel of *C. australis* shows a very weak antimicrobial activity [166]. Similarly, the *C. australasica* peel showed a weak inhibition of acetylcholinesterase (AChE) but showed no cytotoxicity in cell viability assays [333]. The bioactivities of most other species have not been investigated to date. The safety of the *C. glauca* peel extract has not been assessed by the Expert Panel for Cosmetic Ingredient Safety [364].

## 11. Leaf Composition

### 11.1. Phytochemical Composition

Flavonoids reported from *C. australasica* leaves include the following: vitexin (trace), vicenin, poncirin, apigenin 7-neohesperidoside (trace), apigenin 7-*O*-glycoside, kaempferol 3-*O*-rhamnosylglucoside, kaempferol 3-*O*-glucoside, kaempferol 3,7-di-*O*-glycoside, quercetin 3-*O*-monoglucoside, quercetin 3-*O*-diglycoside [365], rutin (0.8 mg/g DW) [327], and naringin (0.7 mg/g FW) [366]. They also contain saponin, triterpenoids, and bound tannins but do not show any antibacterial activity [367].

Grieve and Scora [365] used preparative paper chromatography to identify a number of flavonoids from *C. australis* leaves: the flavone glycosides apigenin 7-neohesperidoside, apigenin 7-*O*-glycoside, acacetin 7-*O*-diglycoside, and luteolin 7-*O*-glucoside and the *C*-glycosylflavones lucenin (trace), a vicenin isomer, saponarin, and rhamnosylvitexin. Other authors have reported low levels of alkaloids in *C. australis* leaves and bark (a single ‘+’ in the screening tests) [368,369].

Kanes et al. [327] and Berhow et al. [326] reported 19 potential phenolic peaks in the leaf tissue of *C. inodora*, of which four were identified: neohesperidin (0.7 mg/g FW), narirutin (0.6 mg/g FW), hesperidin (0.1 mg/g FW), and naringin (0.1 mg/g FW). The total flavanone content (3.96 mg/g) was higher than the flavones and flavonols (1.16 mg/g combined). Additionally, some psoralen compounds were detected but no coumarins. An earlier study on *C. inodora* leaves identified the C-glycosylflavones lucenin and saponarin; the flavanones naringin and poncirin; an uncharacterised flavanone glycoside; the flavone glycoside apigenin 7,4′-diglucoside; and an uncharacterised flavonol glycoside [365]. The leaves also contain moderately high levels of alkaloids (a score of ‘+++’ on the HCl extract but no alkaloids were detected using the prollius extract) [368].

Phytochemicals reported from *C. glauca* leaves include hesperidin (6.1–22.0 mg/g FW), diosmin, poncirin, quercetin 3,7-diglycoside, and narirutin, as well as eight unidentified coumarins [84,326,327,365,366]. Another study identified that the principal flavanone glycoside present was narirutin, which was not the case for another 58 other citrus cultivars studied [84]. Qualitative screening suggests that the leaves contain low levels of alkaloids [368,369], although these have not been characterised.

### 11.2. Volatiles

The leaf volatile profiles of most native Australian *Citrus* species tend to contain unusually large amounts of sesquiterpenoids compared to other citrus. In *C. australasica*, the volatile profile is dominated by bicyclogermacrene (19–28%), germacrene D (2–8%), δ-elemene (0.5–11%), and monoterpenes d-limonene (12–24%) and β-phellandrene (20%) [50]. Katayama et al. [370] and Killiny et al. [353] also reported on the leaf volatiles of *C. australasica* but in less detail.

In contrast, Brophy et al. [50] found that the essential oil distilled from *C. australis* leaves was dominated by α-pinene (68–79% of the total volatile content), with smaller amounts of β-pinene (2–4%), myrcene (3–5%), and d-limonene (2–3%). The essential oil yield was 0.3–0.5%.

The essential oil composition of *C. glauca* leaves is probably the best studied out of all Australian *Citrus* species. Similarly to *C. australis*, α-pinene (60–70%) is the major constituent, followed by nonanal (12%), *p*-cymene, and linalool [46]. Grafted *C. glauca* grown in California also had α-pinene as the dominant volatile (59.5%), followed by β-pinene (11.6%) and two furanoid linalool oxides (5.4% for both). Brophy et al. [50] similarly found α-pinene (24–27%) and β-pinene (12–17%) as the major volatiles, with lower levels of bicyclogermacrene (4–8%), two furanoid forms of linalool (1–15% and 0.8–7%), β-caryophyllene (1–8%), spathulenol (1–6%), and d-limonene (1–2%). In contrast to Hitchcock and Jones [46], Brophy et al. [50] found much more variation in the oil composition from leaves collected at different localities.

The dominance of α-pinene (and β-pinene) is also seen in *C. australis*, but aside from this it is unlike any other Australian or non-Australian *Citrus* species [50]. Additionally, the furanoid linalool oxides are quite rare for citrus, although they have been reported in oil distilled from *C. × aurantium* [371].

The major volatile constituents of *C. inodora* leaf essential oil are germacrene D (4–24%), bicyclogermacrene (1–18%), germacrene B (2–9%), β-caryophyllene (4–20%), δ-elemene (3–5%), β-bourbonene (3–5%), δ-cadinene (2–6%), linalool (2–6%), and phytol (4–9%) [50]. Notably, the volatile profile was almost exclusively dominated by sesquiterpenoids, rather than monoterpenes and monoterpenoids which tend to dominate in other *Citrus* species [372], suggesting that the leaves may not display a typical citrus odour.

Brophy et al. [50] investigated the essential oil composition of a composite sample of leaves from four *C. gracilis* plants, reporting the major volatiles as γ-terpinene (33.8%), (*E*)-nerolidol (20.4%), *p*-cymene (14.8%), and bicyclogermacrene (10.2%). Other constituents included α-pinene, d-limonene, terpinolene, (*E,E*)-farnesene, spathulenol, and phytol. The hydrodistillation essential oil yield was quite low (0.1%), partly due to the high stem/leaf proportion in the samples.

Finally, *C. garrawayi* was the only native Australian *Citrus* species for which Brophy et al. [50] distinguished two chemotypes, based on their leaf essential oil composition. The first chemotype (‘monoterpene chemotype’) was dominated by α-pinene (18–40%), with the monoterpenes camphene (0.2–4%) and myrcene (1–2%) at lower concentrations. It also contained the sesquiterpenes β-caryophyllene (7–13%), α-humulene (2–17%), α- and β-selinene (1–6% in total), bicyclogermacrene (5–8%), globulol (4–10%), and viridiflorol (4–10%).

In contrast, monoterpenes were almost completely absent in the essential oil obtained from several other *C. garrawayi* samples, dubbed the ‘sesquiterpene’ chemotype. It contained β-caryophyllene (17–30%), α-humulene (2–5%), germacrene D (2–4%), α- and β-selinene (0.3–9% total), δ-cadinene (1–8%), globulol (7–10%), viridiflorol (7–10%), and several other oxygenated sesquiterpenes which were not identified (1–9% total).

It is worth noting that while the geographic locations of the chemotypes were not specified, Brophy et al. [50] did collect samples of *C. garrawayi* from two locations: one on the western side of the Cape York Peninsula (Possum Scrub) and one on the eastern side (Turrel Hill). It is possible that these two populations may correspond to the two chemotypes observed.

## 12. Seed Composition

Few studies have characterised the constituents of native *Citrus* seeds. Dreyer [373] tentatively identified limonin and deacetylnomilin from *C. australasica* seeds but did not definitively characterise these compounds. The same authors did not detect limonoids in *C. glauca* seeds. The major fatty acids found in *C. glauca* seed oil are oleic (47.4% of the total), linoleic (36.3%), and palmitic acids (8.1%) [374], while *C. australasica* seed oil contains linoleic (41.0%), oleic (36.6%), and palmitic acids (10.5%), along with 13 other minor fatty acids [374]. One study has investigated the volatile composition of the seeds, reporting the major components as d-limonene (40.4% of the total volatiles), *trans*-sabinene hydrate (35.9%), and γ-terpinene (13.9%) [333]. Screening tests have not found high levels of alkaloids in *C. australis* seeds [171]. *C. glauca* seeds do accumulate chloride [375], which may be linked to salt tolerance. *C. glauca* seed oil is accepted as safe for cosmetic use and is used as an antioxidant, humectant, skin protectant, and emollient [374].

## 13. Commercial Production

As a whole, the Australian *Citrus* species are largely uncommercialised and under-exploited. *C. australasica* has progressed the furthest along the commercialisation path, reaching a small- to medium-scale production.

### 13.1. C. australasica

*C. australasica* was considered one of the top five native species with high commercial potential [376] and has attracted considerable global attention, particularly in the European market [344].

**Figure 19:**
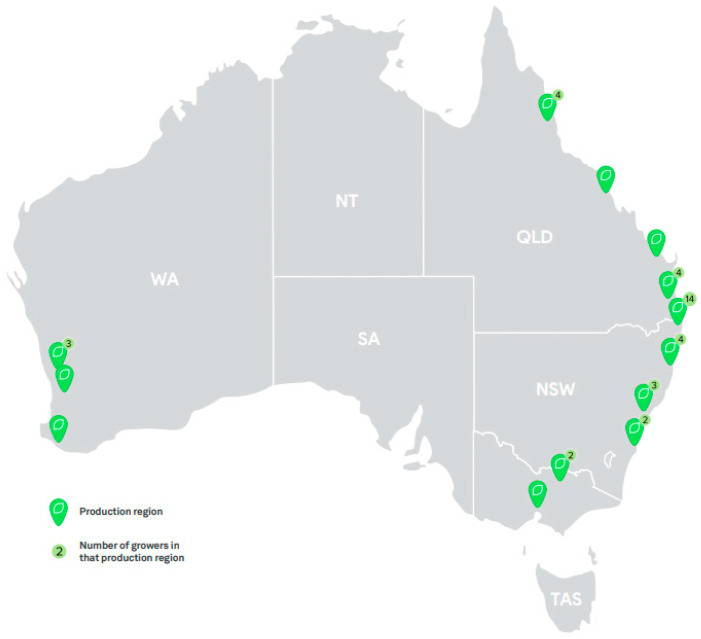
Locations of commercial finger lime (*C. australasica*) growers in Australia in 2022. Numbers indicate individual growers in that location. Reproduced from Glover et al. [172] with permission.

Commercial plantations have been established across Australia, including northern Queensland, southern NSW, and South Australia. There are an estimated 20 major and 50 smaller growers (Figure 19), producing around 103 tonnes annually with a gross value of over AUD 3 million [172]. Farmgate prices range from AUD 47 to 68/kg [172]. Most fruit are sold fresh, although some are processed into jam, chutney, juice, or other novelty uses [376].

The species was imported by the U.S. Department of Agriculture at some point prior to 1911 [377], while the germplasm was also introduced into China and Vietnam more recently (1990s–2000s) [378]. As a result, commercial plantations of *C. australasica* have been established globally, including in Hawai’i [116], California [379], Brazil [380], Japan [330], and Hainan, China [381]. Consequently, establishing the authenticity and provenance of the finger lime (either as whole fruit or dried powder) will likely be of great importance in the future. One recent study by Nastasi et al. [382] demonstrated that near-infrared spectroscopy (NIRS), a rapid, non-invasive, and non-destructive technique, could be used to distinguish between different varieties of Australian finger lime and detect the adulteration of the samples with the Tahitian lime.

Typical commercial crops are planted at a density of 600–800 trees/hectare, with fruiting commencing 2–3 years later and peaking 5–6 years after planting [70]. Trees are either grown ungrafted or sometimes are grafted onto a *C. trifoliata* or Troyer citrange rootstock [383]. Mature trees will typically yield 20 kg annually; half of which is first-grade fruit [70]. Both wild and cultivated trees tend to be alternate bearing (producing a heavy crop one season and a light crop the next). The trees prefer well-drained soil with a pH of 5.0–6.5 [70] and require only 25–30% of the fertiliser used for other citrus cultivars [379]. Some cultivated plants may not achieve the vigour and natural health seen in wild populations. One of the major barriers to widespread commercial production is the requirement for hand harvesting, as the fruit is easily damaged and does not ripen uniformly within the tree. Fruit must be harvested when mature, as they do not continue ripening after picking [70]. The reported shelf life is four weeks if refrigerated at the correct temperature [379].

The fruit extracts are considered safe for use in cosmetic products [349] and have been commercialised by at least one company (Lucas Meyer Cosmetics). Aside from fruit production, *C. australasica* also has uses as a disease-resistant rootstock for other citrus species and as a genetic source of disease resistance, dwarfing, and red-flesh properties in citrus breeding programs [384]. For rootstocks, advanced *C. australasica* × *Poncirus* hybrids are particularly useful, as they show an improved resistance to *Phytophthora* and CTV [145].

### 13.2. C. australis

Some *C. australis* trees were grown as rootstocks for other citrus in the Toowoomba region in the late 19th century [385], but the species has principally only been used as a genetic resource in *Citrus* breeding programs since that time [90]. The highly sticky fruit sap may limit the market for fresh fruit, unless it can be marketed on some novel characteristic which outweighs this downside. Its appearance is the most similar to commercial limes among the Australian species, although it has highly textured skin (Figure 7b).

A survey by Nand [376] of native fruit growers/sellers from southeast Queensland and northeast New South Wales reported a total of 13 growers, 8 plant sellers, and 4 product sellers for *C. australis*. Uses of *C. australis* included fresh fruit, novelty uses, jams, and juices; however, only two growers sold fresh fruit [376]. Most trees were propagated from seeds, as they are slow to strike from cuttings [386].

Another option for commercial uptake is as a value-added flavouring ingredient. Work by Forbes-Smith and Paton [166] showed promising results in this area, while Lim [386] suggested that the fruit was best suited to making drinks, jams, and marmalades and as a lime flavouring and that the peel showed potential for candied peel, spice pastes, or the extraction of an essential oil. However, marmalade made from this species has a slight scum [49], which may be detrimental to commercial products. As suggested by von Mueller [214], selective breeding programs may be useful for improving the fruit quality—both for fresh consumption and processing purposes.

*C. australis* fruit extracts are considered safe for use in cosmetic products [349]. It is currently used in an extract (WildLime Harvest™) commercialised by Lucas Meyer Cosmetics (Knockrow, NSW) for exfoliation purposes.

### 13.3. C. garrawayi

*C. garrawayi* has been grown in citrus collections in the USA since 1915 [15] but has had limited use in breeding programs and has not been widely grown for its fruit anywhere in the world. In breeding, it may be useful as a genetic source of dwarfing, red-flesh properties, and new fragrances [384].

Although its adaptability to conditions outside its natural range is unknown, it has been observed growing in botanic or private gardens in Rockhampton, the Sunshine Coast, and Brisbane. Over two decades ago, Hegarty et al. [171] wrote ‘trials have supported its potential as a successful commercial bushfruit, but there is apparently no chemical information on the fruits;’ and this information is still lacking.

### 13.4. C. glauca

Since the late 19th century, *C. glauca* has been used as a rootstock material for grafting other citrus species [385]. It was introduced into the USA in 1911 and was subsequently distributed to various states in the USA for field trials [73]. While there was no significant uptake among international growers, it continues to be important in citrus breeding programs and is sometimes used as a rootstock [73,200].

Around the turn of the millennium, *C. glauca* was earmarked as a key emerging bushfood species, due to its flavour, processing properties, and plantation potential [387,388]. Richmond et al. [158] estimated the annual production at 13 tonnes in 2019, while Tworney et al. [85] reported a retail price of AUD 25–28/kg in 2009. However, production appears to have slowed in recent years. The main commercial cultivars are the Australian Outback and CR101-13.

The benefits of growing *C. glauca*, summarised from Douglas [42] and other sources, are provided below:

(1) Ease of growing;
(2) Tolerant of frost and heat, so can be grown over a wide climatic area. Cultivated trees have reportedly been grown in all Australian states, including Tasmania [42];
(3) Appealing flavour;
(4) Retains its structure and flavour when frozen;
(5) The fruit is ‘nonbrowning’, unlike most commercial citrus [389];
(6) Wide range of uses. These include puree for food processing, a garnish for fish and chicken, as an ingredient in syrup, jams, curds, chutney, aioli, apple sauce, relish, paste, cordial, cider/liqueur, slush drinks, candied peel/glacé fruit ice cream, and yoghurt. It can also be dried into a powder to use in herb and spice mixtures and yoghurt;
(7) Cosmetic uses—fruit extracts have been used in an Australian manufactured skin cleanser [199,390];
(8) The market demand for seasonal fresh fruit;
(9) The year-round supply of fruit possible in dried or frozen form.

Drawbacks of the species include the following:

(1) The lack of consumer awareness, which can limit the potential market;
(2) The potential for market oversupply (due to a lack of existing demand);
(3) Agronomy is relatively unknown and must be carefully managed;
(4) Labour-intensive, requires further innovation in mechanising processes;
(5) High cost for frozen storage;
(6) Yield can fluctuate depending on the year.

Commercial plantings of grafted *C. glauca* were established near Roma (southwest Qld), Gympie (southeast Qld), Glenrowan (northeast Victoria), southern South Australia, and in Western Australia [42], with the fruit sold on domestic and international markets [200]. By 2017, the industry was approaching the cusp of the transition from niche to commercial production [67]. However, at least some of these plantations (e.g., Roma) have now closed (Jock Douglas, pers. comm.). Most of the current market demand is for a reasonably priced puree, used by gourmet manufacturers [67].

Grafting is generally the recommended propagation method, as some authors have reported that budding is unsuccessful [67]. However, successful budding onto several common rootstocks, including Carrizo, has been anecdotally reported. Certain rootstocks may alter its phenological characteristics. The species is compatible with a wide range of rootstocks, grows best in well-drained soil (to avoid sooty mould and phytophthora), and responds well to fertiliser and irrigation [67]. It takes around 3 years post-grafting for the first harvest and around 12 years to reach the maximum yield of approximately 12 kg/tree [67]. However, large trees may produce >60 kg in exceptional seasons [42]. The harvest period is as short as 3–8 weeks, with fruit deteriorating quickly on the ground [67]. Fruit can be consumed immediately following harvest, refrigerated for 3–4 days, or frozen for longer periods [67].

### 13.5. C. gracilis

There are no record in the literatures of *C. gracilis* being successfully propagated or utilised commercially.

### 13.6. C. inodora

Following its scientific description in 1889, *C. inodora* was not widely propagated or used for several decades. However, after it was imported by the US Department of Agriculture in 1922 [52], it has been utilised in various citrus breeding programs, both in the USA and in Australia [38]. Recently, the US Agricultural Research Service released the hybrid cultivar the ‘Minnie finger lime’, a cross between *C. inodora* and *C. australasica* [361], most notable for its heavy fruiting, compact form, and attractive, almost seedless fruit [391].

One potential barrier standing in the way of commercial adoption is the double-spined trait, which can hinder harvesting and husbandry. Bowman [56] demonstrated that this trait is controlled by a dominant, homozygous nuclear gene, but its expression also relies on one or more independently segregating alleles. Consequently, these spines can be bred out in advanced generation hybrids.

The authors are not aware of any widespread or commercial production of *C. inodora* at present. However, its highly praised flavour and larger fruit may make it a prime candidate for future commercialisation, particularly for spineless varieties.

## 14. Future Research and Directions

### 14.1. Uses of Australian Citrus

There are three main areas where native Australian *Citrus* species may be of particular economic and practical importance: as rootstocks, in citrus breeding programs, and growing the fruit for consumption or processing. There has recently been an increasing research interest in the processing aspect of Australian finger limes (e.g., optimising drying processes [227]). Most of the Australian species readily serve as rootstocks for other *Citrus* varieties, so they could be chosen for their growth characteristics in the target habitat (e.g., dry subtropical and tropical/monsoonal). The main Australian species historically used in breeding programs are *C. australasica*, *C. australis*, and *C. glauca* [90]. However, the other three species (*C. garrawayi*, *C. gracilis*, and *C. inodora*) may also supply beneficial traits. These could include

(1) Drought tolerance (or high rainfall tolerance);
(2) Heat tolerance;
(3) Cold tolerance;
(4) Dwarfing;
(5) Short flowering-to-fruiting periods;
(6) Resistance to *Phytophthora* root rot;
(7) Tolerance to low soil fertility;
(8) Nematode resistance;
(9) Increased levels of potentially beneficial phytochemicals, such as flavonoids.

Additionally, Australian *Citrus* species can serve as a ‘genetic bridge’, to produce desirable traits (such as CTV tolerance, salinity and drought resistance) between other Rutaceae genera (*Atalantia*, *Severinia*, and *Pamburus*) and ‘true’ *Citrus* species [392].

The full genomes of all six native Australian *Citrus* species have recently been assembled, including identifying genes related to disease resistance, antimicrobial peptides, defence, volatile compounds, and acidity regulation [97,104,393]. This will greatly assist existing citrus breeding projects and future commercialisation efforts. However, there are still considerable research gaps that exist. Highlighting this, the best-studied species of the native Australian *Citrus* (*C. australasica)* was recently identified as one of five important but under-investigated species from the *Citrus* genus [394].

### 14.2. Ecology and Morphology

It is highly challenging to study the in situ ecology of the *Citrus* genus (such as seed dispersal and seedling establishment), as few species are still found in their native habitats. Hamilton et al. [395] proposed that native Australian *Citrus* (particularly *C. garrawayi* due to its unique seed morphology and monoembryony) provide an ideal opportunity for studying the connection between the *Citrus* seed morphology and ecological function.

The level of resistance or tolerance which *C. inodora* possesses against CPsV and HLB is another area for future study. In particular, any genetic variability in its resistance to these diseases must be elucidated to aid potential commercial production.

Most aspects of *C. gracilis* are poorly known and require further research. Anecdotal reports by Paul Coats and Michael Saalfeld [55] noted a widely varying morphology, including the tree shape and height (bent 4 m vs. straight 10–12 m), fruit shape (pyriform vs. globose), and number of ovaries (6 vs. 8–9). This suggests that it must be either highly morphologically variable or comprise multiple distinct varieties or even species.

No successful propagation by seeds or cuttings has been reported to date for *C. gracilis*. Laboratory tissue culture methods may be a potential means of propagation [396], but this has not been investigated. Establishing ex situ collections of *C. gracilis* outside of its native range [397,398] may also be important for ensuring the future security of this species. Its disease resistance is unknown, although unpicked fruit are attacked by a black mould.

However, its physiological adaptation to the monsoon conditions of the Northern Territory may prove a valuable genetic resource. The NT citrus industry is currently very small, with 61 ha under production [399]. The use of *C. gracilis* (either grown for its fruit or used in breeding programs) may improve the viability and profitability of the citrus production in this region, while helping future-proof the NT citrus industry in light of changing climatic conditions. Additionally, exporting citrus via the port of Darwin may help reduce shipping costs to major importers in Asia.

### 14.3. Nutritional Value and Chemical Composition

Further research on the chemical/nutritional composition of the fruit is required for almost all native species, particularly for *C. gracilis*, *C. garrawayi*, and *C. inodora*. The fruit of *C. gracilis* reportedly have a resinous flavour; however, they have not been studied scientifically and their composition is completely unknown. For *C. glauca*, sugar profiles and the characterisation of any anti-nutrient components present would be particularly pertinent.

Despite the popularity of *C. inodora* in domestic and international citrus breeding programs, its nutritional composition has not yet been published, and only one group has reported on its flavonoid content. However, this study highlighted an exceptionally high total flavonoid content, suggesting the presence of potentially health-benefiting compounds. Consequently, more detailed investigations into the composition and nutritional value of *C. inodora* fruit should be a priority for future research.

In his 1895 address to the Australasian Association for the Advancement of Science, J.H. Maiden said, ‘We do not yet know the percentage of citric acid in the fruits of our various species of *Citrus* and *Atlantia* [now *C. glauca*]…’ [400]. While this knowledge gap has been partially filled, this statement remains largely true. For example, the citric acid content (along with numerous other compounds) appears to remain unreported for the less ‘popular’ native Australian species (*C. inodora*, *C. garrawayi,* and *C. gracilis*). Consequently, conducting this foundational research on the nutritional composition of native Australian *Citrus* fruit should be a key priority moving forward.

## Figures and Tables

**Table 2:** Change in citrus production between 1961 and 2021 (values in tonnes). Raw data were sourced from FAOSTAT [27].

Citrus Type	1961 Production (t)	2021 Production (t)	% Increase
Oranges	15,976,472	75,567,952	373%
Tangerines, mandarins, clementines	2,835,347	41,950,302	1380%
Pomelos and grapefruits	2,163,741	9,556,999	342%
Other citrus	1,470,554	13,896,889	845%
Total	22,446,114	140,972,142	528%

**Table 4:** Sugar contents of different citrus varieties from various geographic locations (results provided in g/100 g fresh weight).

Citrus Variety	Growing Location	Glucose	Fructose	Sucrose	Reference
Grapefruit	Spain	1.7	2.4	3.5	[239]
Grapefruit	Turkey	2.4	2.3	4.6	[240]
Grapefruit	China	1.1–3.9	1.3–4.8	1.9–6.6	[241]
Grapefruit	China	1.6–2.4	1.6–2.4	2.0–5.5	[242]
Lemon	Spain	0.5–4	2.7–3.0	0.2–2.1	[239]
Lime	Spain	2.4	0.2	0.3	[239]
Mandarin	Spain	0.4–0.6	1.8–2.4	9.5–11.9	[239]
Mandarin	Turkey	2.3–3.5	2.5–3.4	2.6–8.8	[240]
Mandarin	USA	1.0–1.9	1.4–2.4	4.5–7.1	[218]
Orange	Turkey	1.5	1.6	3.5	[240]
Orange	USA	1.0–2.6	1.9–2.7	3.3–5.2	[218]

**Table 7:** The forms and total content of vitamin C found in various citrus varieties (mg/100 g fresh weight).

Citrus Variety	Origin	L-Ascorbic Acid	Dehydroascorbic Acid	Total Vitamin C	Reference
Grapefruit	USA	21.3	2.3	24	[259]
Mandarin, Satsuma	China	30.5	1.2	32	[260]
Mandarin, Ellendale	QLD, Australia	34.0	3.7	38	[261]
Orange Juice	USA	32.6	8.7	41	[259]
Orange	China	57.8	5.0	63	[260]
Orange, Florida	Florida, USA	54.7	8.3	63	[259]
Lemon	QLD, Australia	50.4	23.9	74	[261]
Orange, Navel	California, USA	75.0	8.2	83	[259]

**Table 8:** Levels of B vitamins found in citrus, according to the USDA [218] database. Values are given as µg/100 g on a fresh weight basis.

Citrus Variety	Thiamine (B_1_)	Riboflavin (B_2_)	Niacin (B_3_)	Pantothenic Acid (B_5_)	Pyridoxine (B_6_)	Total Folate (B_9_)
Orange	68	51	425	261	79	25
Lemon	40	20	100	190	80	11
Tangerine	58	36	376	216	78	16
Lime	30	20	200	217	43	8
Grapefruit (white)	40	20	200	283	42	9
RDI (men)	1200	1300	16,000	5000	1700	400
RDI (women)	1100	1100	14,000	5000	1300	400

**Table 10:** The vitamin C content of the *C. australasica* and *C. glauca* pulp reported across different studies. Values are given as mg/100 g on a fresh weight basis. The value for the Tahitian lime is reported at the bottom of the table (grey row) for reference.

Species/Variety	Growing Location	Vitamin C	Reference
***C. australasica***			
Chartreuse	Bundaberg, QLD	23.0 ± 1.3	[284]
Durham’s Emerald	Bundaberg, QLD	34.6 ± 1.3	[284]
Hybrid ‘P1f2-10’	Bundaberg, QLD	31.0 ± 0.5	[284]
Red Champagne	Bundaberg, QLD	53.8 ± 0.7	[284]
Rhyne Red	Bundaberg, QLD	33.0 ± 0.7	[284]
‘Red pulp’ (*sanguinea* type)	Florida, USA	80	[252]
‘White pulp’	Florida, USA	35	[252]
‘Low-seeded, red pulp, large-leaved’ hybrid	Florida, USA	57	[252]
*Sanguinea* type 50–36 cultivar	Florida, USA	115	[252]
‘Green’	QLD, Australia	26 ± 1	[253]
‘Pink’	QLD, Australia	91 ± 2	[253]
‘Red’	Teven, NSW	40.9	[4]
‘Yellow’	Teven, NSW	59.5	[4]
var. *sanguinea*	Australia	82	[3]
Unknown	Lismore, NSW	87.7 ± 5.5	[285]
***C. glauca***	Unknown	188 ± 5	[85]
Tahitian lime	Bundaberg, QLD	19.7 ± 0.2	[284]

**Table 11:** Levels of vitamins and chlorophyll in *C. australasica* and *C. glauca* fruit. Values are given in mg/100 g on a fresh weight basis.

Species	*C. australasica*		*C. glauca*
Cultivar	‘Green’	‘Pink’	
Vitamin E (total)	0.521 ± 0.033	2.360 ± 0.235	0.783 ± 0.194
α-tocopherol	0.517 ± 0.033	2.335 ± 0.233	0.701 ± 0.177
β-tocopherol	-	-	0.081 ± 0.017
γ-tocopherol	0.004 ± 0.0004	0.025 ± 0.002	BDL
δ-tocopherol	BDL	BDL	-
Folate	-	-	0.082
Lutein (provitamin A)	0.401 ± 0.027	0.139 ± 0.011	0.295 ± 0.013
Chlorophyll a	Trace	Trace	Trace
Chlorophyll b	Trace	Trace	1.350 ± 0.044
**References**	[226]	[226]	[226,228,253]

BDL = below detection limit; a dash (-) indicates no data available.

**Table 13:** The main chemotypes reported for the juice volatiles of various *C. australasica* cultivars. Compounds 1, 2, and 3 are the first-, second-, and third-most abundant volatile compounds in each variety. Values are given as percentages of the total volatile composition. The chemotype of the Tahitian lime is reported at the bottom of the table (bold entry) for reference.

Variety	Most Abundant Compound	Second-Most Abundant Compound	Third-Most Abundant Compound	Reference
Collette ^#^	β-caryophyllene (21.4%)	terpinen-4-ol (12.0%)	bicyclogermacrene (10.9%)	[319]
Pink Ice ^#^	sabinene (31.7%)	d-limonene (20.7%)	bicyclogermacrene (14.2%)	[331]
Pink Ice ^#^	terpinen-4-ol (19.3%)	caryophyllene oxide (15.6%)	β-caryophyllene (14.1%)	[319]
Pink Pearl ^#^	d-limonene (32.2%)	γ-terpinene (15.8%)	terpinen-4-ol (15.2%)	[332]
‘Red’ ^#^	bicyclogermacrene (28.0%)	β-bisabolene (24.6%)	viridiflorol (10.9%)	[319]
Unspecified	d-limonene (71.1%)	β-bisabolene (5.2%)	β-phellandrene (3.4%)	[330]
Unspecified ^#^	d-limonene (41.5%)	*trans*-sabinene hydrate (38.4%)	γ-terpinene (14.8%)	[333]
var. *sanguinea* ^#^	d-limonene (45.6%)	sabinene (22.7%)	bicyclogermacrene (10.6%)	[331]
var. *sanguinea* ^#^	d-limonene (66.8%)	γ-terpinene (5.5%)	ledene (4.1%)	[332]
Yellow Sunshine ^#^	bicyclogermacrene (20.3%)	viridiflorol (19.9%)	globulol (14.5%)	[319]
Faustrime (*C. australasica × C. × aurantiifolia*) ^#^	d-limonene (48.3%)	γ-terpinene (10.8%)	α-phellandrene (7.2%)	[319]
Faustrime (*C. australasica × C. × aurantiifolia*) ^#^	d-limonene (35.7%)	β-phellandrene (23.5%)	γ-terpinene (12.6%)	[332]
Faustrime (*C. australasica × C. × aurantiifolia*) ^#^	d-limonene (51.5%)	γ-terpinene (10.0%)	α-bergamotene (7.5%)	[331]
Faustrime (*C. australasica × C. × aurantiifolia*) ^#^	linalyl acetate (18.2%)	d-limonene (11.4%)	citronellol (8.6%)	[334]
**Commercial Tahitian lime** **(*Citrus × latifolia*)** **^#^**	**d-limonene (41.8%)**	**γ-terpinene (14.8%)**	**α-terpineol (10.6%)**	[335]

^#^ The fruit investigated in this study were grown outside of Australia. Shaded rows indicate hybrids or citrus species which are not native Australian citrus species.

**Table 14:** The vitamin C content of the *C. australasica* peel, given as mg/100 g on a fresh weight basis. The value for Tahitian lime is reported at the bottom of the table (grey row) for reference.

Variety	Growing Location	Vitamin C	Reference
Chartreuse	Bundaberg, QLD	26.0 ± 6.8	[284]
Durham’s Emerald	Bundaberg, QLD	34.2 ± 1.5	[284]
Hybrid ‘P1f2-10’	Bundaberg, QLD	49.0 ± 5.9	[284]
Red Champagne	Bundaberg, QLD	42.2 ± 3.8	[284]
Rhyne Red	Bundaberg, QLD	21.3 ± 0.6	[284]
Tahitian Lime	Bundaberg, QLD	36.7 ± 4.6	[284]

## Data Availability

No new data were created or analyzed in this study. Data sharing is not applicable to this article.
